# Oncolytic viruses: advanced strategies in cancer therapy

**DOI:** 10.1038/s41392-025-02343-3

**Published:** 2026-02-05

**Authors:** Danli Xiao, Huarong Zhang, Ye Liu, Yan Li, Gongchu Li, Yunshan Ning

**Affiliations:** 1 School of Laboratory Medicine and Biotechnology, Southern Medical University, Guangzhou, China; 2 Guangdong Provincial Key Laboratory of Immune Regulation and Immunotherapy, Guangzhou, China; 3 Department of Anesthesiology, Beijing Obstetrics and Gynecology Hospital, Capital Medical University, Beijing, China; 4 College of Life Sciences and Medicine, Zhejiang Sci-Tech University, Hangzhou, Zhejiang China

**Keywords:** Tumour immunology, Tumour immunology

## Abstract

Oncolytic viruses (OVs) represent a promising strategy in cancer immunotherapy, as they selectively infect and lyse tumor cells while simultaneously triggering robust antitumor immune responses. By inducing immunogenic cell death, OVs enhance tumor antigen presentation and initiate a systemic immune response, effectively transforming the tumor microenvironment from an immune-suppressive state to an immune-permissive state. In addition to exerting direct oncolytic effects, OVs modulate key tumor-associated biological processes, including tumor angiogenesis and extracellular matrix remodeling, disrupting tumor progression and metastasis. Notably, recent advances have highlighted the therapeutic potential of combining OVs with conventional and emerging cancer treatments, such as chemotherapy, radiotherapy, immune checkpoint inhibitors, adoptive cell therapy, and epigenetic-targeted drugs. These combination strategies demonstrate synergistic effects by improving tumor selectivity, increasing antitumor immunity, and overcoming treatment resistance. Nevertheless, persistent challenges, such as viral dissemination dynamics, therapy resistance, and regulatory complexities, impede the broad clinical implementation of oncolytic virus therapy (OVT). In this Review, we illustrate recent advancements and innovative therapeutic strategies in OVT within the context of contemporary cancer treatment paradigms. First, we outline the historical evolution and key milestones in OVT development. We then discuss the classification of OVs and their multimodal mechanisms that target tumorigenesis, metastasis, disease recurrence, and therapy resistance. Finally, we evaluate the clinical research progress of OVT applications, focusing on their integration with other therapies, analyze the translational barriers hindering clinical implementation, and propose evidence-based future directions for optimizing cancer treatment.

## Introduction

Oncolytic virus therapy (OVT) has emerged as a groundbreaking immunotherapeutic approach that utilizes naturally occurring or genetically engineered viruses to selectively replicate within tumor cells.^1^ Oncolytic viruses (OVs) induce direct tumor cell lysis and simultaneously elicit a systemic antitumor immune response by releasing tumor-associated antigens (TAAs) and recruiting immune cells to the tumor microenvironment (TME).^2^ This dual mechanism of action positions OVT as a highly compelling strategy for cancer treatment, particularly when combined with immunotherapeutic modalities, such as immune checkpoint inhibitors (ICIs) and chimeric antigen receptor (CAR) T-cell therapies. OVT capitalizes on the inherent vulnerabilities of cancer cells, including defective antiviral pathways, which allow viruses to preferentially replicate within malignant tissues.^3^ In contrast to traditional therapies such as chemotherapy and radiotherapy, which frequently have severe side effects, OVT offers the potential for highly specific and less toxic treatment options.^4^ Recent advancements in genetic engineering and its combination with immunotherapy have further improved the safety, efficacy, tumor selectivity, and clinical applicability of OVT.

Currently, OVT is recognized as a rapidly evolving field, drawing increasing clinical and commercial interest. As of 2022, over 400 clinical trials have been conducted to evaluate OVs in various cancers, including melanoma, glioblastoma, lung cancer, and pancreatic cancer.^5^ These trials involve mainly DNA-based viruses, such as herpes simplex virus (HSV), adenovirus, and vaccinia virus (VV), although RNA-based platforms, such as vesicular stomatitis virus (VSV) and measles virus, are also under exploration for their rapid replication and immunogenicity.^6,7^ The commercial potential of OVT is also expanding. The global market was valued at $20.1 million in 2023 and is projected to grow at a compound annual growth rate of 26.9% through 2032.^8^ The approval of four OVs, namely, Rigvir, Oncorine, Talimogene laherparepvec (T-VEC), and Teserpaturev, highlights the increasing clinical validation of this therapeutic approach. Additionally, new candidates, such as Replimune, an engineered HSV expressing a fusogenic protein and granulocyte macrophage colony-stimulating factor (GM-CSF), submitted a Biologics License Application to the U.S. Food and Drug Administration (FDA) in November 2024.^9^ These developments reflect the expanding potential of OVT to transform the landscape of cancer therapy. This review aims to provide a comprehensive and forward-looking evaluation of OVT, encompassing its mechanisms of action, clinical applications, challenges, and emerging perspectives.

## History and milestones of OVs

The concept of using viruses to treat cancer dates back over a century.^10,11^ The earliest documented case of tumor regression following a viral infection occurred in 1904, when a patient with chronic myelogenous leukemia showed a marked reduction in white blood cell count during a flu-like illness.^12^ This observation laid the foundation for subsequent investigations into the oncolytic potential of viruses. In the early 20th century, researchers explored the antitumor properties of wild-type viruses such as flaviviruses, adenoviruses, and hepatitis viruses.^13,14^ These viruses exhibit selective tropism for cancer cells, leading to oncolysis and demonstrating their therapeutic potential. However, most early OVT relied on wild-type virus strains with high titers, which frequently induce viremia and severe infections, sometimes culminating in organ failure or sepsis.^15^ These safety concerns, along with limited knowledge of the mechanisms governing viral tropism for tumor cells, substantially hinder the clinical application of OVT.^14^ By the 1970s and 1980s, enthusiasm for OVT diminished, mainly owing to unresolved challenges and the risks posed by uncontrolled viral replication.^16^

The advent of genetic engineering in the 1990s marked a turning point for the field. This period allowed researchers to manipulate viral genomes precisely, facilitating the transition of OVT from preclinical investigations to clinical applications.^17^ The first generation of genetically engineered OVs was designed to increase tumor selectivity by deleting pathogenic viral genes, thereby restricting viral replication to tumor cells.^18^ For example, thymidine kinase (TK)-deficient HSV showed increased tumor selectivity and reduced systemic toxicity in preclinical models.^19^ The second generation of OVs introduced therapeutic transgenes and genetic modifications to enhance tumor specificity and antitumor immune responses.^20^ For example, viruses were engineered to express cytokines, such as GM-CSF, to recruit immune cells and amplify systemic antitumor immunity.^21,22^ The dual approach of enhancing tumor selectivity while improving immunogenicity represented a significant advancement in OVT, addressing the limitations of earlier virus generations. The third generation of OVs incorporated advanced modifications to evade immune clearance, delivered multiple therapeutic payloads, and synergized with other cancer therapies. For example, Rivadeneira et al. demonstrated that intratumoral delivery of leptin via VV could enhance the effector and memory functions of tumor-infiltrating lymphocytes (TILs) through improving mitochondrial oxidative phosphorylation, thereby enhancing therapeutic efficacy.^23^ Anthony et al. engineered a VV to express a truncated, nonsignaling CD19 protein for tumor-selective delivery, facilitating recognition by CD19-CAR-T cells.^24^ In addition, advances in genome-editing technologies, particularly clustered regularly interspaced short palindromic repeats-associated protein 9 (CRISPR-Cas9), have enabled precise modifications to viral genomes, including the deletion of immune evasion genes and the insertion of tumor-specific promoters, further improving therapeutic specificity and safety.^25–27^

These technological advancements reignited global interest in OVT and catalyzed significant clinical progress. By the early 2000s, the first regulatory approvals for OVs were granted (Table 1). In 2004, Latvia approved Rigvir, a nonpathogenic type 7 human enteric cytopathic orphan virus (ECHO-7), for the treatment of melanoma.^28^ Although Rigvir initially gained recognition, it was later withdrawn from the market because of insufficient clinical evidence. In 2005, China approved Oncorine® (recombinant human type 5 adenovirus H101) for use in combination with cytotoxic chemotherapy for nasopharyngeal carcinoma, marking a critical milestone in the field.^29,30^ Despite these early approvals, the widespread adoption of OVT has been hindered by limited clinical data and concerns regarding its efficacy and safety. In 2015, the FDA approved T-VEC, an attenuated HSV-1 engineered to express GM-CSF, for the localized treatment of recurrent melanoma.^31^ This approval reignited global interest in OVT, highlighting its clinical feasibility and therapeutic potential. Most recently, in 2021, Japan approved Teserpaturev/G47Δ (Delytact), a third-generation HSV-1 derivative, for the treatment of malignant glioma.^32,33^ These findings underscore the increasing validation of OVT as a viable therapeutic modality.

**Table 1:** Currently approved oncolytic viruses worldwide

Name	Virus	Genetic modification	Location	Indication	Results from registry studies
Rigvir*	ECHO-7	Unmodified	Armenia (2016), Georgia (2015), Latvia (2004)	Melanoma	Decreased risk of disease progression with ECHO-7 relative to other experimental immunotherapies, HR 6.67 (P < 0.001).
Oncorine (H101)	Adenovirus	E1B-55K/E3-deletion	China (2005)	In combination with chemotherapy for patients with nasopharyngeal carcinoma	ORR 72.7% in patients receiving H101 plus chemotherapy versus 40.3% with chemotherapy alone; 28.3% of patients had injection site reactions and 9.8% had influenza-like symptoms.
Talimogene (T-VEC)	HSV-1	ICP34.5 and ICP47 gene deletion, GM-CSF insertion	Australia (2016), Europe (2015), Israel (2017), USA (2015)	Unresectable stage IIIB-IV melanoma	DRR 16.3% in patients receiving T-VEC versus 2.1% in those receiving GM-CSF, OR 8.9 (P < 0.001); median OS 23.3 months versus 18.9 months, HR 0.79, 95% CI 0.62–1.00 (P = 0.051).
DELYTACT (G47∆; teserpaturev)	HSV-1	Deletion of ICP34.5, ICP6 and α47 genes	Japan (2021)	R/R glioblastoma following radiotherapy and temozolomide	Median PFS 4.7 months; median OS 20.2 months; grade 3 and 4 adverse events seen in 26.3% and 10.5% of patients, respectively.

*DRR* durable response rate, *GM-CSF*, granulocyte–macrophage colony-stimulating factor, *HSV-1* herpes simplex virus type 1, *ORR* objective response rate, *OS* overall survival, *PFS* progression-free survival, *R/R* residual or recurrent, *T-VEC* talimogene laherparepvec

*Discontinued owing to manufacturing issues in 2019

**Fig. 1:**
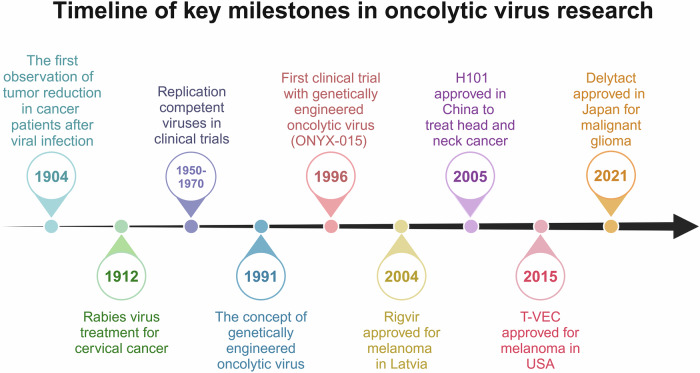
Timeline of key milestones in oncolytic virus research. The therapeutic potential of viral agents in oncology was first recognized in 1904, when spontaneous tumor regression was observed following viral infection. This discovery led to pioneering clinical applications, notably the 1912 use of the rabies virus for cervical cancer treatment. The systematic exploration of replication-competent viruses began in the 1950s--1970s, paving the way for genetic engineering breakthroughs in 1991, which enabled precise modifications for enhanced tumor targeting. A pivotal milestone was reached in 1996 with the first clinical trial of a genetically engineered oncolytic virus, ONYX-015, which drove the clinical translation of OVT. This breakthrough paved the way for key regulatory approvals, including Rigvir (Latvia, 2004) for melanoma, H101 (China, 2005) for head and neck carcinoma, T-VEC (USA, 2015) as the first FDA-approved OV for advanced melanoma, and Delytact (Japan, 2021) for malignant glioma, highlighting OVT’s expanding therapeutic applications. These milestones highlight OVT’s evolution from an experimental concept to a validated cancer therapy, underscoring its growing importance in modern oncology. Created with BioRender.com

In addition to these approved therapies, numerous OVs are currently undergoing clinical trials at various stages (Table 2).^34–36^ The majority of these trials reported promising safety profiles and therapeutic efficacy, further reinforcing the potential of OVT as a transformative approach in cancer treatment. The historical trajectory of OVT, spanning from early observations of tumor regression following viral infection to the advent of sophisticated genetically engineered platforms, highlights the remarkable progress in this field (Fig. 1). The subsequent sections provide an in-depth examination of the mechanisms underlying OVs’ therapeutic actions, their synergistic potential when integrated with combination therapies, and the critical challenges impeding their successful clinical translation.

**Table 2:** Ongoing global clinical trials

Virus type	Name of OVs	Genetic modification	Administration	Tumors	Combination therapy	Status	Clinical trials
HSV	R130	Coding for anti-CD3 scFv/CD86/PD1/HSV2-US11	Intratumoral or intraperitoneal	Advanced solid tumors	None	Early Phase I recruiting	NCT05860374 NCT05961111 NCT05886075
				Head and neck cancer			NCT05830240
				Relapsed/refractory cervical and endometrial cancer			NCT05812677
				Advanced bone and soft tissue Tumors			NCT06171282
				Relapsed/refractory bone and soft tissue tumors			NCT05851456
				Relapsed/refractory ovarian cancer			NCT05801783
	OH2	Deletion of ICP34.5 and ICP47 gene and insertion of GM-CSF	Intratumoral	Solid tumor gastrointestinal cancer	Irinotecan (chemotherapy), HX008 (anti-PD-1)	Phase I/II recruiting	NCT03866525
				Central nervous system tumors	None		NCT05235074
				Solid tumor melanoma	Keytruda (anti-PD-1)		NCT04386967
				Advanced bladder carcinoma	None	Phase II recruiting	NCT05248789
				Non-muscle-invasive bladder cancer		Phase I/II recruiting	NCT05232136
				Advanced liver cancer		Phase I recruiting	NCT05698459
				Melanoma	HX008 (anti-PD-1)	Phase I/II recruiting	NCT04616443
					Chemotherapy	Phase III recruiting	NCT05868707
	RP1	Deletion of ICP34.5 and ICP47 gene and insertion of Fusogenic Protein	Intratumoral	Cutaneous squamous cell carcinoma	None	Phase I/II recruiting	NCT04349436
				Triple negative breast neoplasms	Atezolizumab (anti-PD-L1)		NCT06067061
				Cutaneous squamous cell carcinoma	Cemiplimab (anti-PD-1)	Phase II active not recruiting	NCT04050436
				Melanoma	None	Early Phase I recruiting	NCT06216938
				Advanced solid tumors, melanoma, NSCLC, Non-melanoma skin cancers	Nivolumab (anti-PD-1)	Phase II recruiting	NCT03767348
	RP2 RP3	RP2: Expression of GM-CSF, GALV, and anti-CTLA-4; RP3: Expression of GALV, and anti-CTLA-4 hCD40L, and h4-1BBL	Intratumoral	Refractory metastatic colorectal cancer, pMMR MSS	Atezolizumab (anti-PD-L1), bevacizumab (anti-VEGF)	Phase II active not recruiting	NCT05733611
	RP3	Deletions of ICP34.5 and ICP47 and insertion of GM-CSF and CTLA-4 Antibody, CD40L and 4-1BBL	Intratumoral	Advanced solid tumors	Nivolumab (anti-PD-1)	Phase I active not recruiting	NCT04735978
	T3011	Incorporating the PD-1 antibody and IL-12	Intratumoral	Advanced solid tumors, breast cancer, esophageal neoplasms, HNSCC, non-Melanoma skin cancer, NSCLC, sarcoma	None	Phase I/II recruiting	NCT05205408 NCT05205421
	BS-006	Expression of a bispecific antibody targeting PD-L1 (CD274) and CD3	Intratumoral	Uterine cervical neoplasms	None	Phase I recruiting	NCT05393440
				Solid tumor			NCT05938296
	G207	Deletion of ICP34.5 and UL39 genes	Intratumoral	Recurrent or refractory cerebellar brain tumor	None	Phase I active not recruiting	NCT03911388
	TBI-1401 (HF10)	Deletion of Non-Essential Genes	Intratumoral	Stage III or IV unresectable pancreatic cancer	Gemcitabine, nab-Paclitaxel, TS-1 (chemotherapy)	Phase I active not recruiting	NCT03252808
	C134	Inserting of the PKR evasion gene, IRS1	Intratumoral	Recurrent glioblastoma	None	Phase I recruiting	NCT03657576
	rQNestin	Deletions of γ34.5 and ICP6 genes	Intratumoral	Recurrent malignant glioma	Cyclophosphamide	Phase I recruiting	NCT03152318
	M032	Deletion of ICP34.5 Genes, insertion of IL-12 Gene	Intratumoral	Recurrent/progressive glioblastoma multiforme, anaplastic astrocytoma, gliosarcoma	None	Phase I active not recruiting	NCT02062827
Ad	TILT-123	Encoding for TNF-α and IL-2	Intratumoral	Solid tumor	Gemcitabine	Phase I recruiting	NCT04695327
				Melanoma, HNSCC	Avelumab	Phase I recruiting	NCT05222932
				Metastatic melanoma	None	Phase I active not recruiting	NCT04217473
			Intratumoral or intravenous	Platinum resistant, refractory ovarian cancer	pembrolizumab	Phase I recruiting	NCT05271318
	H101	Deletion E1B-55kD and Partial E3 Gene	Intratumoral	Refractory/recurrent gynecological malignancies	Radiotherapy	Phase II active not recruiting	NCT05051696
				Malignant pleural mesothelioma	PD-L1 inhibitor	Phase I/II recruiting	NCT06031636
				Intrahepatic cholangiocarcinoma	HAIC of FOLFOX (Oxaliplatin, Leucovorin, 5-FU)	Phase IV recruiting	NCT05124002
	Ad-TD-nsIL12	Insertion of Human Non-Secretory IL-12 Gene	Intratumoral	Diffuse intrinsic pontine glioma	None	Phase I recruiting	NCT05717712 NCT05717699
	AdAPT-001	An oncolytic adenovirus armed with a “TGF-β trap”	Intratumoral	Sarcoma, head and neck cancer, TNBC	Checkpoint Inhibitor	Phase II recruiting	NCT04673942
	GM103	Insertion of IL-12 and shVEGF Gene	Intratumoral	Locally advanced, unresectable, refractory, and/or metastatic solid tumors	Pembrolizumab	Phase I/II recruiting	NCT06265025
	CAdVEC	Expression of IL-12 and PD-L1 blocking antibody	Intratumoral	Advanced HER2 positive solid tumors	None	Phase I recruiting	NCT03740256
	ICVB-1042	E1A and E4orf6/7 Dual modifications	Intravenous	Advanced solid tumor	None	Phase I recruiting	NCT05904236
	LOAd703	Encoding TMZ-CD40L and 4-1BBL	Intratumoral	Pancreatic cancer	Gemcitabine, nab-paclitaxel, atezolizumab	Phase I/II recruiting	NCT02705196
	MEM-288	Encoding for human IFN-β and a recombinant chimeric form of CD40-ligand (MEM40)	Intratumoral	Solid tumor	Nivolumab	Phase I recruiting	NCT05076760
	VCN-01	Expression of Hyaluronidase	Intratumoral	Metastatic pancreatic cancer	Nab-paclitaxel, gemcitabine	Phase II recruiting	NCT05673811
VV	GC001	Deletion of TK Genes and insertion of STRIP1 shRNA	Intratumoral	Solid tumor	None	Phase I recruiting	NCT06508307
	ASP1012	Expression of leptin and IL-2	Intratumoral	Solid tumor	Pembrolizumab	Phase I recruiting	NCT06171178
	CF33-CD19	Expression of human CD19 protein	Intratumoral or intravenous	Solid tumor	Blinatumomab	Phase I recruiting	NCT06063317
	Olvi-Vec (GL-ONC1)	Delection of HA, TK and F14.5L gene Insertion of β-Galactosidase, RLuc-GFP and β-Glucuronidase	Intraperitoneal	PRROC, ovarian cancer	Platinum-doublet chemotherapy (carboplatin, cisplatin) and bevacizumab (anti-VEGF)	Phase III recruiting	NCT05281471
	TG6050	Encoding for human IL-12 and anti-CTLA4 antibody	Intravenous	NSCLC	None	Phase I recruiting	NCT05788926
	RGV004	Encoding a bispecific antibody that targets CD19 and CD3	Intratumoral	Relapsed or refractory B-cell lymphoma	None	Phase I recruiting	NCT04887025
	BT-001	Expression of human recombinant anti-CTLA-4 Ab and GM-CSF	Intratumoral	STS, MCC, melanoma, TNBC, NSCLC	Pembrolizumab, [KEYTRUDA®]	Phase I/II recruiting	NCT04725331
	hV01	Deletion of TK and VGF Genes and insertion of IL-21 Gene	Intratumoral	Advanced solid tumor	None	Early Phase I recruiting	NCT05914376
	Pexa-Vec	Expression of GM-CSF	Intratumoral or intravenous	Renal cell carcinoma	Cemiplimab	Phase I/II active not recruiting	NCT03294083
MV	MV-s-NAP	Encoding Helicobacter pylori Neutrophil-activating Protein	Intratumoral	Breast cancer	None	Phase I recruiting	NCT04521764
	MV-NIS	Encoding thyroidal sodium iodide symporter	Intratumoral	MPNST and neurofibromatosis type 1	Platinum chemotherapy; ICIs	Phase I recruiting	NCT02700230
			Intraperitoneal	Platinum-resistant or refractory ovarian, fallopian, or peritoneal cancer	Chemotheapy, bevacizumab	Phase II active not recruiting	NCT02364713
				Recurrent ovarian, primary peritoneal or fallopian tube cancer	None	Phase I/II active not recruiting	NCT02068794
VSV	VSV-IFNβ-NIS	Expression of IFNβ and NIS	Intravenous	Solid tumor, NSCLC, neuroendocrine carcinoma	Pembrolizumab, ipilimumab + nivolumab	Phase I/II recruiting	NCT03647163
			Intravenous	Relapsed/refractory multiple myeloma, acute myeloid leukemia, T and B-cell lymphoma, or histiocytic/dendritic cell neoplasms	Cyclophosphamide, cemiplimab	Phase I recruiting	NCT03017820
			Intratumoral	Stage IV or recurrent endometrial cancer	Ruxolitinib, phosphate; radthearpy	Phase I active not recruiting	NCT03120624
Orthopoxvirus	CF33-hNIS	Expression of the human sodium iodide symporter	Intratumoral or intravenous	Solid tumor, cholangiocarcinoma, bile duct cancer	Pembrolizumab	Phase I recruiting	NCT05346484
	CF33-hNIS-antiPDL1	Expression of the human sodium iodide symporter and Anti-PD-L1 scFv	Intratumoral	Metastatic TNBC	None	Phase I active not recruiting	NCT05081492
Maraba virus	MG1MA3	Transgenic expression of MAGEA3 tumor-associated antigen and mutation in M and G proteins	Intravenous	Advanced or metastatic solid tumor	MAGEA3-expressing, adenoviral vaccine	Phase I active not recruiting	NCT02285816
Reovirus	Pelareorep	None	Intravenous	TNBC	Retifanlimab	Phase II recruiting	NCT04445844
			Intratumoral	Metastatic breast cancer	Paclitaxel, avelumab (anti-PD-1)	Phase II active not recruiting	NCT04215146

*HSV* herpes simplex virus, *GM-CSF* granulocyte-macrophage colony-stimulating factor, *PD-1* programmed cell death protein 1, *PD-L1* programmed death-ligand 1, *NSCLC* non-small cell lung cancer, *HNSCC* head and neck squamous cell carcinoma, *VEGF* vascular endothelial growth factor, *CTLA-4* cytotoxic T-lymphocyte-associated protein 4, *TNBC* triple negative breast cancer, *STS* soft tissue sarcoma, MCC merkel cell carcinoma, *HER2* human epidermal growth factor receptor 2, *HAIC* hepatic arterial infusion chemotherapy, *CAR-T* chimeric antigen receptor T-Cell, *PRROC* platinum resistant or refractory ovarian cancer, *hNIS* human sodium iodide symporter, *TCR* T-cell receptor, IRS1 insulin receptor substrate 1, *STRIP1* striatin-interacting protein 1

The type of tumor and the progress of clinical trials with the relative code are indicated as reported on https://clinicaltrials.gov/, accessed on 17 September 2024

## Classification of OVs

OVs are categorized as single- or double-stranded DNA or RNA viruses on the basis of their genetic composition and structural properties. DNA viruses generally possess larger genomes, enabling extensive genetic modifications to optimize therapeutic efficacy and regulate immune responses while preserving viral replication capacity.^1^ In contrast, RNA viruses have relatively smaller genomes but exhibit rapid replication and the ability to cross the blood‒brain barrier, rendering them especially effective in targeting central nervous system malignancies.^37^ However, the restricted genome size of RNA viruses constrains their capacity to accommodate large transgenes. Moreover, compared with DNA viruses, RNA viruses are characterized by increased genetic instability and elevated mutation rates.^38^ Table 3 provides a list of OVs commonly used in clinical settings.

**Table 3:** The dominate types of oncolytic viruses

Virus species	Genome	Genome size	Methods of entry	Replication site	Advantages	Example
Herpesvirus	dsDNA	150 kb	HVEM, nectin-1, nectin-2	Nucleus	Large genome to insert large fragments and multiple transgenes; drug to shut-off	T-VEC, G47∆
Adenovirus	dsDNA	26-45 kb	CAR, CD46, VCAM-1	Nucleus	Feasibility of manufacturing high viral titers; ease of genome manipulation; inherently potent lytic activity	H101, ONYX-015
Vaccinia virus	dsDNA	~190 kb	Receptor-mediated endocytosis	Cytoplasm	Fast, efficient spreading virus; high-speed life cycle; up to 40kd large gene fragment insertion; enough knowledge due to smallpox	Pexa-Vec, GL-ONC1
Reovirus	dsRNA	~23.5 kb	Sialic acid, JAM1	Cytoplasm	Good adaptability for intravenous injection; natural selectivity towards various cancer cells; displays no dose-limiting toxicity	Reolysin®
Coxsackievirus	ss(+)RNA	~15 kb	CAR, ICAM-1, DAF	Cytoplasm	Not pathogenic in humans; no integration into host genome; direct activation of tumor-specific lymphocytes and macrophages	CVA21
Poliovirus	ss(+)RNA	7.5 kb	CD155	Cytoplasm	Can cross BBB; does not infect normal cells; non-pathogenic in humans; tumor specific	PVSRIPO
Newcastle disease virus	ss(-)RNA	15 kb	Sialic acid	Nucleus	Naturally targets tumor cells; low immunogenicity; high safety; induces strong anti-tumor immune response	rNDV-IL2-TRAIL
Measles virus	ss(-)RNA	16 kb	SLAM, CD46	Nucleus	Low immunogenicity; high immune escape ability in the body; naturally targets tumor cells; strong immune response	MV-NIS
Vesicular stomatitis virus	ss(-)RNA	11 kb	LDLR	Cytoplasm	Well understood; associated with relatively mild disease; can be produced in high titers for viral production.	VSV-IFNβ-NIS

*dsDNA* double-stranded DNA, *dsRNA* double-stranded RNA, *ssRNA* single-stranded RNA, *VCAM-1* vascular cell adhesion molecule-1, *HVEM* herpesvirus entry mediator, *CAR* coxsackie adenovirus receptor, *JAM-A* junctional adhesion molecule A, *ICAM1* intercellular adhesion molecule 1, *DAF* decay-accelerating factor, *SLAM* signaling lymphocytic activation molecule, *LDLR* low-density lipoprotein receptor, *nAbs* neutralizing antibodies

### Herpes simplex virus

As a double-stranded DNA virus, HSV has a genome size of ~120–152 kb, encoding more than 70 genes, which makes it one of the most extensively utilized OV platforms.^39^ HSV-1, in particular, serves as a cornerstone in OV research and therapeutic development. It is the first virus to be engineered as a recombinant OV vector and the first OV to receive FDA approval for cancer treatment.^40,41^

HSV has several advantages as an oncolytic platform: (1) it replicates rapidly in tumor cells and can infect a wide range of cancer cell types; (2) its large genome allows for extensive genetic modifications,^42^ including the insertion of multiple therapeutic transgenes to enhance antitumor efficacy;^43,44^ (3) antiviral drugs such as acyclovir can effectively neutralize HSV when necessary, providing additional safety measures;^45–47^ and (4) modifying HSV glycoproteins can improve its tumor-targeting capabilities, thereby increasing specificity and therapeutic outcomes.^48^ These characteristics make HSV-1 an ideal candidate for genetic engineering in cancer therapy. Common genetic modifications include the deletion of viral genes necessary for replication in normal cells but redundant in cancer cells, such as TK, ICP34.5 (crucial for viral replication in neurons), ICP6 (which encodes the large subunit of HSV-1 ribonucleotide reductase), and ICP47.^49–52^ Deleting ICP47 reduces neurotoxicity and prevents HSV-1 from inhibiting major histocompatibility complex class I (MHC-I) antigen presentation, thereby augmenting host immune responses and facilitating immunogenic cell death (ICD) within the TME.^49,53^

A notable oncolytic HSV-1 therapy is T-VEC, the first HSV-1-based OV approved by the FDA. It was engineered by deleting the γ-34.5 and α47 genes and inserting the GM-CSF gene.^42^ These modifications increase the tumor selectivity and immunogenicity of T-VEC.^54–58^ Clinical studies have shown that T-VEC is safe and well tolerated when administered intralesionally and exhibits significant anticancer efficacy in multiple solid tumors, including breast cancer, gastrointestinal cancer, and melanoma.^59–61^ Importantly, T-VEC increases CD8^+^ T-cell infiltration while reducing the number of CD4^+^ regulatory T cells (Tregs) in the TME, indicating its dual role as an oncolytic agent and an immunotherapy that enhances systemic antitumor immunity.^62^ Compared with GM-CSF monotherapy, T-VEC consistently showed superior durable response rates and improved overall survival (OS) in patients with advanced melanoma.^31^

In addition to T-VEC, other genetically engineered oncolytic HSV-1 variants have demonstrated promising results. G47Δ, a triple-mutant HSV-1 derived from the second-generation OV G207, contains deletions in both copies of the γ-34.5 gene, the α47 gene, and an inserted β-galactosidase gene.^63,64^ G47Δ has shown broad-spectrum efficacy against various solid tumors in preclinical models, including glioma, breast cancer,^65^ prostate cancer,^64,66^ schwannoma,^67^ nasopharyngeal carcinoma,^53^ hepatocellular carcinoma,^68^ malignant peripheral nerve sheath tumor,^69^ and thyroid carcinoma.^70^ Notably, G47Δ efficiently targets and eliminates glioblastoma stem cells, which contributes to tumor recurrence and resistance to conventional therapies.^71,72^ In 2017, the Daiichi Sankyo Company designated G47Δ as an orphan drug to treat malignant glioma. In 2021, G47Δ became the first OV approved in Japan for glioma therapy.^73^ Similarly, rQNestin34.5, an HSV-1 mutant expressing ICP34.5 under the control of the nestin promoter, exhibited potent antitumor effects in glioma models both in vitro and in vivo.^74^ This design leverages nestin, a tumor-specific marker that is overexpressed in glioma cells to increase replication specificity and therapeutic efficacy.

Although most oncolytic HSV strains are developed by deleting the ICP34.5 gene, studies suggest that retaining ICP34.5 may enhance viral replication and oncolytic effects in malignant tumors that have residual type I interferon (IFN) signaling or are in a potential IFN-dependent antiviral state.^75^ Researchers have proposed the use of microRNA response elements in the 3’ untranslated region of HSV to regulate ICP34.5 gene expression, thereby ensuring selective viral replication in cancer cells and improving its replication efficiency.^76^ This microRNA genetic switch offers a potential multifunctional strategy to restrict OV replication in normal cells.

### Adenovirus

Adenovirus (Ad) is a nonenveloped, icosahedral, double-stranded DNA virus with a genome size ranging from 26–45 kb, depending on the serotype. Among the 57 identified serotypes, Ad2 and Ad5 of subgroup C are the most widely utilized oncolytic adenoviruses (OAds).^77,78^ Ad enters cells through endocytosis^79^ or receptor-mediated pathways, such as binding to the coxsackievirus and adenovirus receptor or integrins,^80,81^ subsequently releasing its DNA into the nucleus for replication via the host’s transcriptional machinery. The adenoviral genome remains episomal, avoiding integration into the host genome, thereby enhancing safety by mitigating the risks of insertional mutagenesis.^82^ Therefore, its broad tissue tropism and high gene delivery capacity make Ad a popular tool in OVT.^83^

The replication of Ad in normal cells depends on early genes such as E1A and E1B.^21,84,85^ However, these genes are dispensable in tumor cells, where dysregulated signaling pathways compensate for the functions of E1A and E1B. For example, E1A induces the dissociation of E2F from the retinoblastoma (pRB) complex, thereby driving cells into S phase and activating downstream viral genes.^86^ To achieve tumor-selective replication, many OAds incorporate mutations or deletions in the E1A and E1B genes. For example, the deletion of 24 base pairs in E1A (E1A-d24) prevents viral replication in normal cells but enables replication in pRB-deficient tumor cells.^87,88^ Similarly, E1B deletions can enhance tumor selectivity by exploiting defective p53 signaling, a hallmark feature of many cancer cells.^89^ H101 (Oncorine), the first approved recombinant OAd in China, carries deletions in E1A and E1B, which enable selective replication in p53-deficient tumor cells.^6,90^ It was approved in 2005 following successful clinical trials for nasopharyngeal carcinoma. Onyx-015, an OAd with E1B deletion, has demonstrated safety and efficacy in clinical trials for head and neck cancer, pancreatic cancer and ovarian cancer.^78^ These pioneering developments in adenoviral engineering have paved the way for further advancements in OAds.

Despite their therapeutic potential, the low expression of coxsackievirus and adenovirus receptors on many tumor cells poses a challenge for efficient adenoviral infection.^80^ To address this limitation, capsid modifications have been investigated. For example, inserting an arginine-glycine-aspartic acid (RGD) motif into a fiber knob enhances binding to integrin αvβ3, which is highly expressed in tumor cells.^88^ This modification improves viral entry and tumor tropism.^91^ Similarly, alternative receptors, such as CD46 (upregulated in colorectal and breast cancers) and desmoglein (frequently overexpressed in malignant epithelial tumors), can be targeted through fiber protein engineering.^92,93^ Other capsid modifications, such as polylysine modification in CRAd-S-pk7, enhance viral penetration into tumor cells. CRAd-S-pk7, a conditionally replicative OAd driven by a tumor-specific survivin promoter, has shown potential in treating high-grade gliomas in phase I clinical trials.^94^

To further increase tumor-killing efficiency, Ad can be engineered to carry therapeutic genes. These include genes encoding proteins such as TNF-related apoptosis-inducing ligand (TRAIL) and adenoviral death protein (ADP), both of which directly induce apoptosis in tumor cells.^95,96^ Prodrug activator genes, including bacterial cytosine deaminase and TK from HSV, have also been incorporated into OAds.^97–99^ These genes convert nontoxic prodrugs such as 5-fluorocytosine or ganciclovir into cytotoxic agents, specifically at the tumor site.^100^ Importantly, these cytotoxic agents can diffuse into adjacent uninfected tumor cells via gap junctions, amplifying the therapeutic effect and achieving a “bystander killing” effect.

OAds not only directly mediate oncolysis but also elicit a strong antitumor immune response. Upon lysis, tumor cells release TAAs and damage-associated molecular patterns (DAMPs), which activate the host immune system. To further potentiate immune activation, OAds are increasingly being modified to express immunomodulatory proteins.^82^ For example, GM-CSF, CD40 ligand, and IFN-I are frequently incorporated into the Ad genome to amplify both innate and adaptive immune responses.^101–104^ These immune-stimulating properties enable OAds to establish persistent antitumor immune memory, improving long-term prognosis and preventing tumor recurrence.

Clinical trials have been conducted to evaluate the efficacy and safety of OAds.^6^ For example, CG0070, an Ad5-based vector regulated by an E2F-responsive promoter and encoding GM-CSF, has shown promising efficacy in treating nonmuscle-invasive bladder cancer.^105,106^ In a phase III clinical trial, CG0070 achieved a clinical response rate of approximately 45% in patients with deficiencies in apoptosis-related gene expression.^107^ Similarly, TILT-123, an OAd expressing IL-2 and TNF-α, is undergoing trials for multiple solid tumors.^108^

### Vaccinia virus

VV, a member of the *Orthopoxvirus* genus of the *Poxviridae* family, possesses a linear double-stranded DNA genome of approximately 190–200 kb. Historically, VV has played a crucial role in the eradication of smallpox and has gained attention as a promising OV.^109^ The large genome size of VV allows for the incorporation of multiple therapeutic transgenes. Its inherent tumor selectivity mitigates the risk of insertional mutagenesis, as VV replicates exclusively in the cytoplasm without integrating into the host genome.^110–112^ VV preferentially infects and replicates in tumor cells while sparing normal cells, exhibiting natural tumor tropism. This property, along with its capacity to replicate within the hypoxic TME, makes VV especially effective against solid tumors that are resistant to conventional therapies.^113^ VV exhibits a rapid and highly lytic replication cycle of approximately eight hours. These features underscore the potential of VV as a powerful oncolytic agent. Moreover, VV displays effective and stable bloodborne transmission, conferring a distinct advantage over other OVs, such as Ads and HSVs,^114,115^ which are more prone to immune clearance in circulation. The VV employs mechanisms such as apoptotic mimicry for cell entry, bypassing the need for specific receptors, which expands its applicability to various tumor types.^116,117^

To increase safety and tumor specificity, VV has undergone extensive genetic modifications. One common modification is the deletion of the TK gene, which is essential for viral DNA synthesis in normal cells. In cancer cells, increased intracellular TK levels facilitate selective viral replication.^118^ TK gene deletion increases tumor specificity while minimizing off-target effects.^119,120^ Additionally, deletion of the vaccinia growth factor (VGF) gene and the introduction of mutations in the A56R and F14.5 L genes, which encode hemagglutinin and a secretory signal peptide, respectively, further improve the tumor selectivity of VV and attenuate its activity in normal tissues.^109,121,122^

Several VV strains have been engineered to incorporate transgenes that boost their antitumor activity. For example, the incorporation of cytokines such as GM-CSF into the VV genome stimulates the immune system to target tumors more efficiently. JX-594 (Pexa-Vec), a strain-derived VV, incorporates GM-CSF and harbors a deletion in the TK gene.^123^ JX-594 has demonstrated promising results in preclinical and clinical studies, including phase I and II trials for advanced hepatocellular carcinoma. These trials confirmed tumor-specific replication and enhanced antitumor immunity.^124^ Notably, a randomized phase II trial by Heo et al. revealed that high-dose JX-594 improved OS compared with low-dose JX-594 in patients with advanced hepatocellular carcinoma. Although a subsequent phase III trial in patients with advanced hepatocellular carcinoma failed to show a survival benefit,^125^ JX-594 remains a critical step in OVT development.

In addition to JX-594, other VV-based platforms have exhibited promising therapeutic potential. TG6002, a Western Reserve strain-derived VV, contains deletions in both the TK and viral ribonucleotide reductase genes, along with the integration of the cytosine deaminase/uracil phosphoribosyltransferase (FCU1) gene.^126^ TG6002 selectively converts the noncytotoxic prodrug 5-fluorocytosine (5-FC) into its cytotoxic form, 5-fluorouracil, within tumor cells. This strategy amplifies tumor-specific cytotoxicity while minimizing collateral damage to normal tissues. TG6002 is currently undergoing phase I/II clinical trials for the treatment of brain cancer (NCT03294486).^127^ Another example is GL-ONC1, a Lister strain-derived VV engineered with three insertion mutations (Ruc-GFP, β-glucuronidase, and β-galactosidase).^128^ In a phase I trial involving patients with advanced peritoneal carcinomatosis, GL-ONC1 demonstrated a favorable safety profile, with no dose-limiting toxicities (DLTs) observed and clear evidence of tumor-specific activity.^129^ Similarly, ACAM2000, a TK-positive VV strain, has been evaluated in patients with advanced solid tumors (stage III or IV).^130^ This treatment was well tolerated across multiple dosing regimens and demonstrated promising antitumor activity, including substantial tumor size reduction or complete eradication in some patients.

Despite its potential, the clinical application of VV in OVT faces several challenges. VV originates from the smallpox vaccine strain, resulting in widespread pre-existing immunity within the population, which may limit its therapeutic efficacy.^131^ Neutralizing antibodies induced by prior smallpox vaccinations or initial OV administration can accelerate viral clearance, thus reducing the duration of therapeutic benefit.^132^ Strategies to circumvent these limitations include combining VV-based therapies with ICIs or modifying the viral envelope to evade immune recognition. Another promising approach involves leveraging VV to enhance the tumor immune microenvironment. For example, the incorporation of IL-10 has been shown to prolong viral persistence by dampening antiviral immunity while preserving antitumor immune responses.^133^ Combination strategies that integrate VV with other immunotherapeutic agents, such as programmed death-1/programmed death ligand 1 (PD-1/PD-L1) inhibitors, have demonstrated considerable potential in increasing overall antitumor efficacy.^134^ Our research team has developed GC001, an innovative oncolytic VV platform engineered to express small hairpin RNA (shRNA) targeting striatin-interacting protein 1 (STRIP1), a gene involved in antiviral immunity. This modification enhances tumor specificity and cytotoxicity by circumventing antiviral defenses in tumor cells, thereby promoting viral replication within the TME. Currently undergoing a phase I clinical trial (NCT06508307), GC001 has shown potential for expanding therapeutic options for cancer patients while minimizing off-target effects.

### Reovirus

Reovirus, also known as respiratory and enteric orphan virus, is a nonenveloped double-stranded RNA virus with a 60–80 nm icosahedral structure. Reovirus primarily enters host cells through receptor-mediated endocytosis by binding to junctional adhesion molecule A (JAM-A), its principal receptor. Reovirus is naturally present in the respiratory and gastrointestinal tracts of mammals, including humans, without inducing significant illness.^135^ However, reovirus exhibits a natural tropism for tumor cells with an activated RAS signaling pathway, which is frequently dysregulated in various cancer types.^136^ Reovirus is categorized into three distinct serotypes, namely, Type 1 Lang, Type 2 Jones, and Type 3, which includes the Abney and Dearing strains. Notably, the Type 3 Dearing strain, commonly called T3D, has been developed into the therapeutic agent Reolysin®.

Pelareorep, an unmodified wild-type reovirus, preferentially replicates in cancer cells, inducing cell lysis and triggering immune responses through cytokines that activate immune cells, thereby contributing to tumor cell death.^137^ Early-phase clinical trials confirmed its safety and tolerability upon intravenous administration, with no DLTs observed.^137^ In the initial phase I trial (REO-001) involving patients with advanced solid tumors, intratumoral injections of Pelareorep demonstrated tumor responses in 37% of patients, with adverse effects primarily limited to Grade 2 or lower.^138^ In 2015, the FDA granted the Reolysin® orphan drug designation for treating ovarian cancer, pancreatic cancer, and glioblastoma multiforme. In 2017, it received fast track designation for metastatic breast cancer.^75^ However, as a monotherapy, Pelareorep has shown limited efficacy, leading to a shift in clinical strategies toward combination therapies involving chemotherapy, radiotherapy, or ICIs.

A series of phase II trials have assessed Pelareorep in combination with standard chemotherapies across several cancer types, including pancreatic adenocarcinoma, ovarian cancer, metastatic non-small cell lung cancer (NSCLC), colorectal cancer advanced melanoma, and metastatic breast cancer.^125,139–142^ Although most trials did not show significant improvement in progression-free survival (PFS) or OS compared with chemotherapy alone, Pelareorep combined with paclitaxel showed a notable OS benefit in metastatic breast cancer patients, with the median OS improving from 10 months in the chemotherapy-only group to 17.4 months in the combination therapy group.^143^ Similarly, a phase II trial in patients with advanced melanoma reported a median OS of 10.9 months and a median PFS of 5.2 months when Pelareorep was combined with carboplatin and paclitaxel.^144^ However, these results should be interpreted cautiously because of the limited cohort size and insufficient statistical power to detect differences in OS.^145^

In recent studies, the immune-stimulatory properties of Pelareorep have been explored in combination with ICIs. Pelareorep enhances antitumor immunity by upregulating the expression of proinflammatory cytokines, such as interleukin-12 (IL-12), GM-CSF, and IFN-γ, while downregulating the expression of protumoral factors, such as IL-8 and vascular endothelial growth factor (VEGF).^136,146,147^ These changes shift the TME toward a proinflammatory state and promote T-cell infiltration. A phase Ib study combining Pelareorep with pembrolizumab (anti-PD-1) and chemotherapy for advanced pancreatic adenocarcinoma demonstrated increased CD8^+^ T-cell infiltration and upregulation of the cytotoxic T lymphocyte (CTL)-attracting cytokines C-X-C motif chemokine ligand 10 (CXCL10) and CXCL11.^148^ This immune modulation correlated with prolonged survival in certain patients, suggesting that Pelareorep may augment the efficacy of ICIs. However, a subsequent phase II trial (NCT03723915) evaluating Pelareorep and pembrolizumab in pancreatic cancer patients failed to meet its primary endpoint, leading to early termination. Similarly, interim results from ongoing phase I/II trials evaluating Pelareorep in combination with atezolizumab (anti-PD-L1) for advanced gastrointestinal cancers and hormone receptor-positive/human epidermal growth factor receptor 2 (HER2)-negative breast cancer have yielded encouraging findings.^149^ In advanced pancreatic ductal adenocarcinoma, three patients treated with Pelareorep and atezolizumab achieved partial responses, with no significant safety concerns reported.^150,151^ In breast cancer patients, Pelareorep combined with atezolizumab resulted in increased TILs and a favorable shift in CelTIL scores, a metric correlated with therapeutic response.^143^ In particular, mutations within σ1 have been introduced to prevent proteolytic cleavage of σ1 by breast cancer-associated proteases, which disrupt binding to sialic acid, thereby restoring infectivity in the σ1 mutants.^152^ These innovations suggest that a new and exciting era of reovirus research is emerging.

Preclinical studies have further confirmed the ability of Pelareorep to induce apoptosis, autophagy, and immunomodulation in tumor cells. Its natural tropism for tumor cells with a dysregulated RAS signaling pathway, along with its ability to stimulate robust antitumor immunity, positions Pelareorep as a promising candidate for combination therapies. However, challenges remain, including inconsistent efficacy as a monotherapy and the need for larger, well-powered clinical trials to validate its combination strategies. Future studies should focus on optimizing dosing regimens and identifying predictive biomarkers to maximize the clinical benefit of Pelareorep.

### Other OVs

In addition to the four common OVs mentioned, other viruses, particularly single-stranded RNA viruses, which can be classified into positive-strand and negative-strand single-stranded RNA viruses, have shown promising potential in OVT.

Positive-strand single-stranded RNA viruses, including poliovirus, coxsackievirus, and seneca valley virus (SVV), are members of the *Picornaviridae* family and replicate in the cytoplasm without a DNA phase, thereby eliminating the risk of insertional mutagenesis during infection.^153^ This characteristic makes them safer for therapeutic applications and facilitates genetic manipulation for enhanced oncolytic activity.^154^ For example, coxsackievirus enters cells by binding to decay-accelerating factor (DAF) and intercellular adhesion molecule 1 (ICAM-1), which are frequently upregulated in cancers such as melanoma, multiple myeloma, and breast cancer.^155,156^ Coxsackievirus A21 (CVA21, Cavatak) has demonstrated good tolerability and preliminary efficacy in multiple clinical trials.^157–160^ In a clinical trial for nonmuscle invasive bladder cancer (NCT02316171), intravesical administration of CVA21 led to increased tumor surface bleeding and inflammation, with some patients exhibiting complete tumor regression.^159^ In melanoma treatment, intravenous administration of CVA21 in phase II clinical trials (NCT01227551 and NCT01636882) resulted in a PFS rate of 32.9% and a durable response rate of 21.1%.^158^ Additionally, combining CVA21 with ICIs such as pembrolizumab has shown significant efficacy.^160^ Another coxsackievirus subtype, CVB3, has shown efficacy in preclinical models of NSCLC, particularly in A549 lung adenocarcinoma xenografts that are resistant to radiation and epidermal growth factor receptor (EGFR) tyrosine kinase inhibitors.^161^ CVB3 exploits the overexpression of DAF in NSCLC cells, inducing caspase-mediated apoptosis and subsequent oncolysis while circumventing complement-mediated cytotoxicity.^162,163^ SVV is a nonpathogenic, nonenveloped RNA virus from the *Picornaviridae* family with a natural tropism for neuroendocrine tumors, particularly small cell lung cancer (SCLC).^164^ Its ability to infect neuroendocrine tumors stems from specific pathways that facilitate tumor targeting. A significant advantage of SVV is its inherent resistance to hemagglutination, enabling efficient intravenous delivery by preventing premature viral clearance and improving its ability to target the tumor site following systemic administration.^165^ In a phase I clinical trial, 30 patients with advanced solid tumors, including six with SCLC, received SVV-001 intravenously.^166^ The therapy was well tolerated, with no DLTs observed. Notably, viral clearance was observed in all patients and was correlated with the development of antiviral antibodies. In SCLC patients, in vivo intratumoral viral replication was confirmed, with peak viral titers exceeding 10^3^-fold higher than the administered dose. One patient with chemoresistant SCLC exhibited a remarkable PFS of 10 months, highlighting the virus’s potential in this challenging tumor type. Despite its potential, clinical trial results have been variable, indicating the necessity for further optimization.^167^ Poliovirus, owing to its high neurovirulence in human motor neurons, has been modified by replacing its internal ribosome entry site (IRES) with that of human rhinovirus type 2, thereby reducing its pathogenicity. This modification allows the virus to specifically target malignant tissues expressing CD155, a receptor frequently overexpressed in glioma cells, thereby enhancing its potential as a glioma therapy.^168^

Negative-strand single-stranded RNA viruses, such as measles virus (MV) and Newcastle disease virus (NDV), are also gaining traction in OVT. These viruses have a larger virion size and complete their life cycle within the cytoplasm, disseminating infection through cell‒to-cell fusion. This mechanism leads to the formation of multinucleated syncytia, which induce tumor cell death and trigger immune responses against the tumor. MV enters host cells via receptors such as signaling lymphocyte-activation molecule (SLAM), CD46, or Nectin-4,^169^ whereas NDV infects host cells through sialic acid binding.^170^ These viruses complete their life cycle in the cytoplasm, spreading infection via cell-to-cell fusion, which leads to the formation of multinucleated syncytia and subsequent cell death. The favorable safety profile and absence of DLTs make MVs potential candidates for OVT. A promising variant is MV-NIS, an oncolytic MV encoding the sodium iodide symporter (NIS). MV-NIS has been shown to induce immune responses against resistant ovarian cancer in a phase I/II trial (NCT02068794) and has been deemed safe for intraperitoneal administration.^171^ In a separate clinical trial (NCT00450814) involving patients with relapsed or refractory multiple myeloma, MV-NIS resulted in one patient achieving complete remission, with several others showing partial remission.^172^ These results suggest that MV holds significant potential, particularly when it is used in genetically modified forms to enhance its therapeutic efficacy.

NDV exerts oncolytic effects through both direct and indirect mechanisms. The direct mechanism involves tumor-selective oncolysis, where NDV infects tumor cells and induces cell death through multiple apoptotic pathways.^173,174^ This involves the formation of multinucleated syncytia through the fusion of infected cells, the activation of both intrinsic and extrinsic apoptosis pathways, and the activation of the mitogen-activated protein kinase (MAPK) cascade along with the endoplasmic reticulum (ER) stress response.^175,176^ The fusogenic properties of the virus, which are mediated by the viral fusion protein, cause fusion among adjacent infected cells, resulting in extensive syncytium formation. Syncytium formation enhances apoptotic cell death and viral spread, thereby improving bystander killing of tumor cells.^177,178^ Additionally, the presence of NDV hemagglutinin-neuraminidase and fusion proteins is crucial for stable syncytial formation, and their ratio significantly influences this process.^179,180^ Mutations in these proteins, such as a lysine-to-arginine substitution in the fusion protein, can create hyperfusogenic NDV strains that enhance the targeting of heterogeneous tumors.^177^ In addition to direct oncolysis, NDV infection triggers the release of cytokines such as IFN-α, IFN-β, and TNF-α, which activate the nuclear factor kappa-B (NF-κB) signaling pathway, contributing to apoptosis.^175,177,179^ Interestingly, NDV-induced apoptosis may occur independently of viral replication and involves mitochondrial pore activation, caspase-8 engagement, and viral nucleocapsid protein expression. The indirect approach also involves both the innate and adaptive immune systems, where infected tumor cells release TAAs and danger signals, leading to the activation of antigen-presenting cells (APCs) and the recruitment of immune cells such as T cells, dendritic cells (DCs), and natural killer (NK) cells.^181,182^ NDV enhances the expression of adhesion molecules (ICAM-1, LFA-3) and MHC molecules on tumor cells, promoting immune recognition and targeting.^183^ Additionally, NDV is capable of replicating in tumor cells that are resistant to apoptosis, as well as in tumor cells that lack oxygen or are resistant to IFN.^184^

However, relying solely on its inherent viral capabilities, NDV does not provide the required specificity and therapeutic efficacy for widespread clinical application. Therefore, recombinant engineering and modification of NDV are necessary to increase its targeting ability and efficacy against cancer. For example, rNDV-IL2-TRAIL, which expresses IL-2 and TRAIL, induces apoptosis and significantly enhances antitumor activity.^185^ rNDV-B1/Fas incorporates the human Fas gene, a well-known inducer of apoptosis.^186^ Fas signaling is critical in both the intrinsic and extrinsic apoptosis pathways. The presence of Fas in rNDV-B1/Fas enhances apoptosis, thus significantly improving the oncolytic properties of the virus. In preclinical syngeneic mouse models, rNDV-B1/Fas exhibited significant tumor regression and improved survival, demonstrating its potential as an effective oncolytic agent for cancer therapy. Moreover, Numpadit et al. engineered a recombinant NDV designed to release IFN-γ and target melanoma cells. Wei et al. generated a recombinant NDV Italian strain carrying the cHAb18 gene (rNDV-18HL), which is capable of inhibiting the invasion and migration of HCC cells through the binding affinity of the mouse-human chimeric HAb18 antibody for the tumor-associated antigen CD147.^187,188^ Notably, excessive expression of foreign genes can affect NDV replication, posing a challenge in the development of recombinant NDV as a cancer therapeutic.^189^

## The antitumor mechanism of OVs

### Direct oncolytic activity

**Fig. 2:**
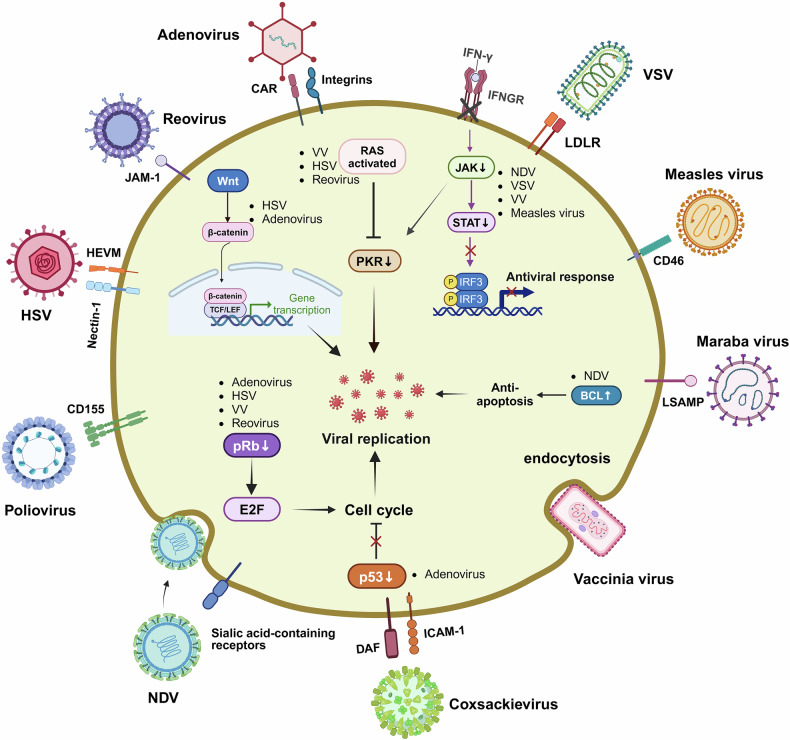
Pathways, receptors, and mechanisms used by OVs to target cancer cells. OVs exploit tumor-specific vulnerabilities to selectively infect and lyse cancer cells. These viruses take advantage of overexpressed extracellular receptors, such as CAR, ICAM-1, DAF, CD155, CD46, and sialic acid-containing receptors, which are abundantly present on the surface of tumor cells. These receptors facilitate viral entry, allowing OVs to initiate infection and replicate within the tumor. Additionally, OVs exploit dysregulated intracellular signaling pathways, including Ras, PKR, p53, and E2F, which are frequently altered in cancer cells. These disruptions enhance viral replication and promote oncolysis by bypassing normal host defense mechanisms. Furthermore, defective antiviral responses in tumor cells, such as impaired IFN-γ signaling, further support viral replication, enabling OVs to efficiently propagate within the tumor microenvironment. By leveraging these tumor-specific characteristics, OVs preferentially target cancer cells while sparing healthy tissues, making them a promising strategy for targeted cancer therapy. CAR coxsackievirus-adenovirus receptor, DAF decay-accelerating factor, HSV herpes simplex virus, HVEM herpesvirus entry mediator, ICAM-1 intercellular adhesion molecule-1, LDLR low-density lipoprotein receptor, NDV Newcastle disease virus, VV vaccinia virus, VSV vesicular stomatitis virus, LSAMP limbic system associated membrane protein, E2F E2 transcription factor, PKR protein kinase R. Created with BioRender.com

OVs selectively target and eliminate cancer cells while sparing healthy tissues. This tumor-specific targeting is driven by two main mechanisms: the recognition of tumor-specific surface receptors and the exploitation of unique vulnerabilities in the TME. Consequently, OVs preferentially infect and replicate in cancer cells, thereby disrupting their cellular functions and ultimately inducing cell death.^190^ Their ability to recognize and infect tumor cells is mediated by interactions with tumor-specific surface receptors and intracellular signaling pathways (Fig. 2).^191^ OVs interact with distinct receptors, which are often overexpressed on tumor cells but exhibit minimal expression on normal cells.^92,192,193^ For example, OAd utilizes receptors such as coxsackievirus and adenovirus receptors, integrins, and CD46 for cell entry,^194^ whereas MV employs CD46, and HSV-1 targets NECTIN or herpesvirus entry mediators.^195^ Other OVs, such as NDV and VV, lack specific attachment receptors and instead rely on endocytosis for cellular entry, thereby broadening their applicability to diverse tumor types.^175,196^ This receptor diversity highlights the adaptability of OVs in targeting heterogeneous tumor populations. By exploiting specific receptor‒ligand interactions, OVs achieve tumor selectivity while minimizing their cytotoxic effects on normal cells.

Once inside tumor cells, OVs exploit dysregulated signaling pathways and altered metabolic states within the TME to replicate effectively.^197^ Tumor-specific features, such as hyperactive Ras signaling, hypoxic conditions, and uncontrolled proliferation, create a favorable environment for viral replication.^198–200^ For example, Ras signaling activation enhances nucleotide metabolism and upregulates transcription factors essential for viral gene expression, amplifying OV replication within tumor cells.^201–204^ Tumor hypoxia suppresses antiviral responses and enhances the replication of certain OVs adapted to low-oxygen environments. This tumor-specific targeting capability minimizes off-target effects, optimizing the therapeutic index while enhancing the safety and efficacy of OVs in cancer therapy. OVs subsequently hijack the host cell’s biological machinery to facilitate their replication. This interference disrupts essential cellular processes, including protein and nucleic acid synthesis, leading to the dysfunction of critical organelles such as the nucleus, mitochondria, and ER. The resulting cellular stress induces apoptosis or necrosis. For example, recombinant NDV R2B-GFP induces a loss of mitochondrial membrane permeability in 4T1 and B16-F10 cells, triggering apoptosis and amplifying its therapeutic effect.^205^ After replication, OVs release newly formed viral progeny from lysed tumor cells. These progeny infect neighboring cancer cells, initiating a self-sustaining cycle of infection and oncolysis.^206,207^ Capsid proteins contribute to cellular destruction by disrupting membrane integrity, increasing viral release and spread.^208^ This cycle continues until tumor cells are depleted or immune clearance mechanisms are activated, making OVs highly effective tools for reducing the tumor burden.

The TME plays a key role in enhancing OV replication and activity. Owing to impaired antiviral defenses, tumor cells are unable to mount effective immune responses against viral infections. In normal cells, viral infections activate innate immune pathways, including type I IFN signaling, which involves Janus kinase (JAK), signal transducer and activator of transcription (STAT), and interferon regulatory factors (IRFs).^209,210^ These pathways promote antiviral gene expression, induce apoptosis in infected cells, and trigger proinflammatory cytokine production to limit viral spread. However, tumor cells often downregulate key components of these pathways, such as retinoic acid-inducible gene I (RIG-I), IRF3, and IRF7, increasing their susceptibility to OV infection.^1^ Once cancer cells undergo lysis and die, OVs are released, spreading viral replication to adjacent cells.

In addition to these natural mechanisms, genetic engineering has been widely applied. Tumor-specific promoters (TSPs), such as human telomerase reverse transcriptase (hTERT) and midkine promoters, ensure that viral gene expression and replication primarily occur in malignant cells.^211–213^ By placing OV genes under the control of these promoters, viral replication is enhanced within tumor cells, improving therapeutic precision and minimizing harm to healthy tissues. Genetic modifications can also enhance OV selectivity by impairing the antiviral responses of tumor cells (Table 4). For example, VSV-D51 was engineered to reduce its inhibitory effects on IFN signaling, ensuring selective replication in IFN-deficient tumor cells while sparing normal cells with intact IFN signaling pathways.^214^

**Table 4:** Important pathways targeted by oncolytic viruses and their functional mechanisms

Specific OV tumoricidal pathways	Functional mechanisms
p53 Pathway	Many cancers inactivate the p53 tumor suppressor pathway through mutations in p53 or loss of upstream/downstream regulators. OVs such as adenovirus, reovirus, and parvovirus preferentially target p53-null or mutant cells. OVs exploit p53 pathway defects by degrading p53 or avoiding apoptotic signals, replicating in p53-deficient tumor cells.
Wnt Signaling Pathway	Wnt pathway activation is common in cancers, particularly gastrointestinal tumors. OVs like CRAds exploit Wnt signaling defects to selectively replicate in tumor cells with defective Wnt signaling. The oncolytic HSV bM24-TE uses a synthetic promoter that activates viral replication in colorectal cancers with APC mutations.
IFN Signaling Pathway	OVs exploit defects in the interferon (IFN) pathway often found in tumor cells. While normal cells use IFNs to trigger antiviral responses, many tumors have downregulated IFN signaling, making them more permissive to viral replication. OVs like VSV, NDV, and reovirus selectively target these defects for enhanced oncolytic activity.
Anti-apoptotic Pathways	Many cancer cells evade apoptosis through overexpression of anti-apoptotic proteins like Bcl-xL. OVs, including adenovirus, HSV, and vaccinia virus, have evolved mechanisms to evade apoptosis in normal cells while utilizing these pathways to replicate and kill tumor cells, which often overexpress anti-apoptotic proteins.
pRb Pathway	The retinoblastoma (pRb) pathway is frequently dysregulated in cancers, making cells more susceptible to oncolytic viruses like adenovirus and HSV. These OVs exploit pRb pathway defects by using modified viral proteins (such as E1A) to selectively replicate in pRb-deficient tumor cells, resulting in tumor cell lysis.
PI3K/Akt/mTOR Pathway	OVs like NDV can target the PI3K/Akt/mTOR pathway to induce autophagy and overcome resistance to first-line treatments such as cisplatin and paclitaxel. This pathway is important for regulating hypoxia-inducible factors (HIFs) and promoting viral replication and tumor cell death.

Another promising strategy to increase OV selectivity involves miRNA-based modifications. Tumor cells often display distinct miRNA expression profiles, with certain miRNAs being downregulated in malignant tissues.^215^ Inserting miRNA response elements into the 3’ untranslated region of viral genes enables OVs to exploit the differential expression of specific miRNAs between normal and tumor cells.^216^ This enables OVs to preferentially replicate in tumors where specific miRNAs are downregulated while reducing replication in normal cells that express these miRNAs at relatively high levels. For example, engineered OAds such as OA-4MREs have demonstrated enhanced replication and oncolysis in glioma cells through the incorporation of miR-124, miR-128, miR-146b, and miR-218.^217^ This strategy increases the therapeutic precision of OVs, which target cancer cells while sparing normal tissues. Moreover, OVs can be engineered to deliver tumor suppressor miRNAs (e.g., miR-143 and miR-34a) directly into tumor cells.^218^ These miRNAs can inactivate key oncoproteins, such as kirsten rat arcomaviral oncogene homolog (KRAS), triggering apoptosis and suppressing tumor growth. This approach has been shown to enhance both oncolytic efficacy and tumor specificity. For example, in osteosarcoma and HCT116 xenografts, oncolytic VSV and OAd carrying miR-143 induced apoptosis and significantly suppressed tumor growth. Similarly, engineering OVs to carry oncogenic miRNAs (e.g., miR-21) can reduce viral replication in normal cells while maintaining high oncolytic activity in tumors overexpressing these miRNAs.^219^

Recent studies have highlighted the role of RNA modification pathways in regulating OV replication and enhancing oncolytic efficacy. For example, the HSV-1 protein ICP0 manipulates RNA modification pathways in tumor cells, particularly by downregulating the m6A “writer” methyltransferase like 14 (METTL14), reducing antiviral responses and enhancing viral replication.^220^ These findings support the idea that RNA modifications play a key role in optimizing viral oncolysis. Furthermore, epigenetic regulation of the TME influences OV efficacy. A recent study revealed that bromodomain and extraterminal domain (BET) inhibitors enhance viral replication by inhibiting insulin-like growth factor 2 mRNA binding protein 3 (IGF2BP3)-induced NETosis, providing a promising approach to overcome resistance in gliomas.^221^ These studies underscore the potential of combining miRNA-based strategies with epigenetic modifiers to increase the precision and effectiveness of OVT.

### Activation of antitumor immunity

OVs play a crucial role in activating the host’s innate immune system, which triggers an adaptive immune response that targets tumors.^1^ Various OVs, including OAd, HSV, coxsackievirus, VV, and NDV, induce ICD to varying degrees. The ICD is essential for triggering antitumor immune responses and promoting immune memory.^222–226^ ICD, a cornerstone of the antitumor immune response induced by OVs,^227^ encompasses several forms of cell death, including apoptosis, necrosis, ferroptosis, autophagic cell death, and pyroptosis.^228–230^ During ICD, tumor cells release TAAs and tumor-associated neoantigens (TANs), both of which are crucial for activating adaptive immunity.^231–233^ Simultaneously, the release of pathogen-associated molecular patterns (PAMPs) and DAMPs triggers the innate immune system and inflammatory responses. These molecules act as alarm signals, alerting the immune system to cellular stress or damage in the TME.^234^

DAMPs, including heat shock proteins (HSPs), high mobility group box 1 protein (HMGB1), adenosine triphosphate (ATP), and calreticulin, play key roles in orchestrating the innate immune response. These molecules are released or exposed on the tumor cell surface during OV-induced ICD and are recognized by pattern recognition receptors on immune cells such as DCs, NK cells, and M1-like macrophages.^235^ This interaction facilitates the recruitment, activation, and maturation of innate immune cells, which then produce proinflammatory cytokines and chemokines, amplifying the immune response.^234,236^ For example, calreticulin, an ER-associated chaperone, translocates to the tumor cell surface during ICD. It then counteracts CD47-mediated “don’t eat me” signals, enhancing tumor cell phagocytosis by DCs and macrophages.^237,238^ Additionally, ATP acts as a chemoattractant, promoting the infiltration of DCs into the TME. HMGB1 binds to Toll-like receptor-4 on DCs, driving their maturation and the upregulation of costimulatory molecules necessary for effective antigen presentation. Beyond innate immune activation, OV infection induces cellular stress responses, such as ER stress and genotoxic stress, in tumor cells. These stresses lead to the production of reactive oxygen species (ROS) and antiviral cytokines, including type I IFNs.^236^ Type I IFNs are especially critical for enhancing NK cell cytotoxicity and promoting antigen presentation by DCs, thereby linking innate and adaptive immunity.^239,240^ However, excessive IFN production can upregulate the expression of immunosuppressive checkpoint molecules, such as PD-L1. These findings underscore the potential of combining OVs with ICIs to increase therapeutic efficacy.^241^

Following OV-induced ICD, the release of TAAs, TANs, and DAMPs recruits APCs, such as DCs, to the infection site.^242^ APCs bridge innate and adaptive immunity by presenting processed tumor antigens on major MHC molecules to T cells.^243,244^ This process is essential for activating and expanding tumor-specific CTLs. Upon maturation, DCs migrate to secondary lymphoid organs, such as lymph nodes, where they present MHC-peptide complexes to CD4^+^ and CD8^+^ T cells. This process, along with costimulatory signals from molecules such as CD40, CD80, and CD86 and cytokines such as IL-12 and type I IFNs, drives the activation, proliferation, and differentiation of T cells into tumor-specific effector T cells.^222,245–248^ Activated T cells return to the tumor site, are guided by chemokines, and exert cytotoxic effects by recognizing tumor cells presenting the corresponding antigens, subsequently releasing perforin and granzymes.^246,249,250^ These molecules induce apoptosis in tumor cells, enhancing overall antitumor responses. The activation of tumor-specific CTLs is particularly important for generating systemic and long-term immunity, preventing tumor recurrence and metastasis.

**Fig. 3:**
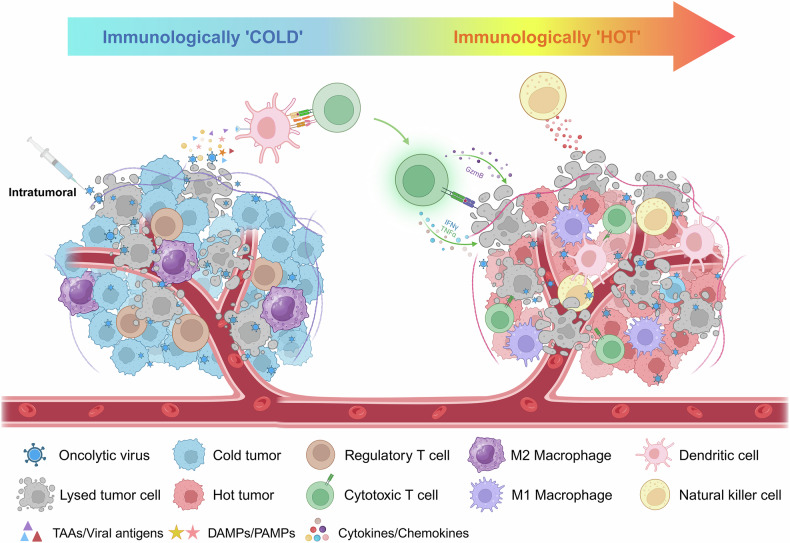
OV-induced antitumor immunity transitions from “cold” to “hot” tumors. OVs infect tumor cells, leading to cell lysis and the release of TAAs, viral antigens, DAMPs and PAMPs into the TME. This process activates the immune system, stimulating the release of cytokines and chemokines that recruit immune cells, including dendritic cells, macrophages, natural killer cells, and T cells, into the TME. Initially, the TME is “cold”, with minimal immune infiltration and a limited antitumor response. OVs help transform the TME into a “hot” environment characterized by increased infiltration of immune cells, including cytotoxic T cells, M1 macrophages, and natural killer cells. This shift enhances tumor cell recognition and eradication, promoting antitumor immunity. TAAs tumor-associated antigens, DAMPs damage-associated molecular patterns, PAMPs pathogen-associated molecular patterns, TME tumor microenvironment. Created with BioRender.com

OVs not only stimulate immune responses but also actively remodel the TME, overcoming its inherent immunosuppressive characteristics. Tumors are often in a “cold” state characterized by limited immune cell infiltration; high levels of Tregs, myeloid-derived suppressor cells (MDSCs), and M2-like macrophages; and elevated production of immunosuppressive cytokines such as transforming growth factor-β (TGF-β) and IL-10.^251,252^ Hence, targeting these immunosuppressive elements may increase the efficacy of OVs. For example, a VV encoding the TGFβRII inhibitor gene induced partial tumor regression in a mouse model.^253^ OVs convert immunologically “cold” tumors into “hot” tumors, fostering an environment that supports effective immune cell infiltration and activation. Multiple mechanisms contribute to this transformation (Fig. 3). For example, OVs reprogram macrophages from an immunosuppressive M2 phenotype to a proinflammatory M1 phenotype, increasing their tumoricidal activity.^254^ They also reduce Tregs and MDSCs in the TME, alleviating immune suppression.^255^ OVs upregulate MHC class I and II molecules and costimulatory molecules on APCs, enhancing antigen presentation and facilitating robust adaptive immune responses.^256^ Additionally, localized secretion of type I IFNs and other cytokines enhances the recruitment of effector immune cells, including CD8^+^ T cells and NK cells.^225^ For example, oHSVs have been shown to decrease the number of MDSCs and Tregs in the spleen, reversing systemic immune suppression.^257^ This shift promotes tumor clearance and prevents recurrence by establishing immune memory.

A major advantage of OVT lies in its capacity to establish long-lasting antitumor immunity. OVs promote robust T-cell responses, preventing tumor recurrence and metastasis. Tumor-selective OVs release TAAs and TANs, which are processed by APCs and presented to CD8^+^ T cells, generating memory T cells capable of recognizing and eliminating residual or recurring tumor cells. For example, the M1 virus, an oncolytic reovirus, disrupts immune tolerance in the TME, enhances CD8^+^ T-cell activity, and establishes durable immune memory in poorly immunogenic tumor models.^47^ An oncolytic VV encoding IL-7 and IL-12 can lead to complete tumor regression in murine models and confer resistance to rechallenge.^258,259^ Similarly, OVs expressing IL-12 or GM-CSF promote sustained antitumor immunity by driving T-cell proliferation and activation. These effects extend beyond the primary tumor, offering systemic protection against metastases and tumor recurrence.

To enhance their capacity for ICD induction and immune activation, OVs have undergone extensive genetic engineering. These modifications aim to increase the secretion of immunostimulatory molecules, improve immune cell recruitment, and counteract immunosuppressive mechanisms. GM-CSF is a cytokine that promotes DC maturation and antigen presentation.^260^ OVs, including VV, HSV, VSV, and MV, have been engineered to express GM-CSF, enhancing immune cell recruitment and activation.^261–264^ For example, GM-CSF-expressing HSV (T-VEC) strains exhibit significant tumor regression and systemic immune responses, including effects on distant uninjected tumors.^265^ Chemokines also play crucial roles in the TME by attracting immune cells to tumor sites.^266^ Owing to this ability, OVs have been engineered to express chemokines, increasing their antitumor efficacy, particularly for converting “cold” tumors into “hot” tumors. Intratumoral administration of CXCL11-expressing VV promotes the accumulation of adoptive T cells in tumor tissues, prolonging the survival of tumor-bearing mice.^267,268^ However, an oncolytic VSV engineered to express CXCL9 did not show enhanced antitumor activity compared with that of the control virus,^269^ indicating that careful selection or strategic combination of these cytokines is necessary for developing more efficient OVs. In addition, IL-2 can activate both innate and adaptive immunity, primarily through effector T cells and Tregs, and has been shown to be effective in cancer therapy.^270^ However, IL-2 has a short half-life and requires frequent administration to maintain bioavailability, which limits its clinical application.^271^ Consequently, OVs are engineered to encode IL-2, ensuring its localized expression in tumors and enhancing their antitumor activity. Oncolytic HSV encoding IL-12 (oHSV-IL-12) exhibited significant antitumor activity against hepatic tumors and was more effective at preventing tumor rechallenge.^272^ This antitumor efficacy was associated with elevated IL-12 and IFN-γ expression, which induced increased infiltration of CD4^+^ and CD8^+^ T cells in the TME.

### Impact on the tumor ECM

The extracellular matrix (ECM) is a highly dynamic and complex structure composed of proteins, including collagen, fibronectin, and laminin, as well as nonprotein components such as hyaluronic acid (HA). The ECM plays a crucial role in regulating tumor invasion, metastasis, and drug resistance.^247,273,274^ The ECM constitutes up to 60% of the tumor mass in solid tumors and is synthesized predominantly by activated cancer-associated fibroblasts (CAFs). These stromal cells are key contributors to the immunosuppressive TME, actively facilitating tumor progression and immune evasion.^275^ The rigid and dense ECM serves as both a physical barrier and an immune shield, restricting leukocyte infiltration and hindering the penetration of therapeutic agents into tumor nests.^274,276^ In pancreatic ductal adenocarcinoma (PDAC) and lung cancer, the density and structure of the ECM isolate T cells within the tumor stroma prevent their infiltration into the tumor nests and contribute to the formation of an “immune desert”.^276,277^ The deposition and remodeling of ECM components, such as collagen and HA, further exacerbates this effect, positioning ECM components as promising targets for cancer therapy. OVs effectively dismantle structural barriers, impeding immune cell infiltration into the TME.^275^

CAFs are the primary architects of the ECM and are responsible for producing and remodeling its components to promote tumor growth and confer therapy resistance.^278^ CAFs secrete various growth factors, cytokines, and chemokines, such as TGF-β, IL-6, and CXCL12, which drive cancer cell proliferation, survival, and migration.^279–281^ CAFs also modulate the behavior of immune and stromal cells within the TME, reinforcing its immunosuppressive properties. For example, CAF-derived TGF-β suppresses antitumor immunity and reprograms stromal cells to promote tumor progression.^282^ Moreover, CAFs contribute to therapeutic resistance by increasing ECM rigidity, which acts as a physical shield against chemotherapy and immunotherapy, and by secreting factors that inhibit immune cell activity.^281,283^ Interestingly, reciprocal signaling between tumor cells and CAFs enhances tumor progression and OV replication.^284^ For example, TGF-β secreted by tumor cells increases the susceptibility of CAFs to OV infection by suppressing their antiviral defenses. In turn, CAFs produce fibroblast growth factor 2 (FGF2), which downregulates RIG-I expression in tumor cells, making them more permissive to OV replication. This cross-talk creates a niche of OV-sensitive cells within the TME, thereby enhancing the therapeutic potential of OVs.

To overcome ECM-mediated physical and immunosuppressive barriers, OVs can be engineered to degrade ECM components and selectively target CAFs.^285–288^ For example, VSV, which exhibits a natural tropism for CAFs, has demonstrated efficacy in preclinical models of PDAC by selectively infecting and lysing CAFs.^284^ In addition to targeting tumor cells, certain OVs, such as OAds, are designed to simultaneously target both CAFs and tumor cells. For example, OAds equipped with fibroblast activation protein (FAP)-specific ligands can selectively infect FAP^+^ CAFs, disrupting their tumor-promoting activities and remodeling the ECM.^289^ Genetically modified OVs can degrade crucial ECM components, increasing drug delivery and facilitating immune cell infiltration. HA, a critical ECM component associated with tumor aggressiveness and poor prognosis, is a prime target for ECM-modulating strategies.^290^ The accumulation of HA in the TME creates a dense matrix that restricts lymphocyte infiltration, reducing the efficacy of immune-based therapies, including ICIs, CAR-T cells, and TILs.^291^ By degrading HA, OVs can convert the TME into a more conducive environment for immune activation and therapeutic intervention. A notable example is OVV-Hyal1, an oncolytic VV engineered to express hyaluronidase.^292^ This virus degrades HA in solid tumors, facilitating viral dissemination, improving chemotherapeutic drug penetration, and increasing leukocyte infiltration. OVV-Hyal1 has demonstrated improved efficacy in combination therapies, significantly augmenting the therapeutic effects of CAR-T cells, peptide-based therapies, and ICIs. Similarly, the OAd ICOVIR17, which expresses hyaluronidase, showed remarkable efficacy in glioblastoma models when combined with PD-1 blockade, leading to the recruitment of tumor-associated proinflammatory macrophages and augmenting T-cell cytotoxicity both locally and systemically.^286^ In PDAC, innovative strategies have been developed to increase OV efficacy by modulating ECM-related interactions. For example, a chemically engineered OAd (oAd/DCN/LRP-PEG-NT) conjugated with neurotensin peptide and polyethylene glycol (PEG) demonstrated significant ECM-degrading efficacy.^293^ This virus disrupted the Wnt signaling pathway, enhancing oncolytic activity against PDAC. These modifications highlight the potential of OVs to overcome ECM-mediated resistance and enhance therapeutic efficacy.

The immunosuppressive properties of the ECM hinder immune cell infiltration and restrict the diffusion of therapeutic agents. OVs armed with ECM-degrading enzymes, including collagenase, matrix metalloproteinases, and hyaluronidase, show substantial potential in overcoming these challenges.^294^ For example, an OAd expressing relaxin, a peptide hormone that softens the ECM, significantly improved the penetration of therapeutic monoclonal antibodies in PDAC models.^295^ Relaxin expression disrupted collagen-rich barriers within the TME, facilitating deeper infiltration of immune cells and therapeutic agents. The ECM-modulating effects of OVs extend to the tumor vasculature. Excessive ECM deposition around blood vessels contributes to abnormal vascular architecture and impairs drug delivery. By remodeling the ECM, OVs can normalize the tumor vasculature, enhancing drug distribution and oxygenation within the tumor core.^296^ This process not only enhances the efficacy of conventional therapies but also creates a more immunogenic TME.

Despite promising results from ECM-targeting OVs in preclinical and early-phase clinical studies, several challenges remain. For example, ECM degradation requires precise regulation to mitigate potential risks, such as tumor metastasis or excessive inflammation. Additionally, the heterogeneous composition of the ECM across different tumor types necessitates tailored OV designs for optimal therapeutic outcomes. Future research should focus on leveraging advanced genetic engineering tools to develop multifunctional OVs capable of simultaneously degrading the ECM, targeting CAFs, and augmenting immune responses.

### Destruction of the tumor vasculature

Persistent abnormal angiogenesis is a hallmark of most tumor types.^297,298^ Under hypoxic conditions within the TME, the sustained activation of the “angiogenic switch” drives continuous neovascularization and increases the expression of proangiogenic factors, including VEGF.^66^ VEGF and other angiogenic factors not only drive vascularization but also contribute to immune evasion by creating physical and biochemical barriers that restrict immune cell infiltration.^298^ Consequently, disrupting the tumor vasculature and inhibiting neovascularization are crucial for effective tumor suppression.^299^

Numerous studies have demonstrated that OVs possess potent antiangiogenic properties through diverse mechanisms.^300–303^ OVs can directly infect and lyse tumor-associated vascular endothelial cells, resulting in a substantial reduction in tumor blood flow, severe hypoxia, and extensive tumor necrosis. For example, VSV infection has been shown to trigger extensive neutrophil infiltration, resulting in thrombosis, ischemia, and subsequent tumor cell death due to perfusion loss.^304^ Similarly, JX-594 (Pexa-Vec) specifically targets endothelial cells in the tumor vasculature, disrupting the VEGF and FGF-2 signaling pathways. This disruption results in vascular leakage, severe hypoxia, and ultimately extensive tumor necrosis.^305^ Unlike traditional antiangiogenic therapies that aim to normalize the tumor vasculature, OV-mediated vascular disruption facilitates the systemic delivery of OVs and immune cells into the TME. In another example, recombinant iNDV3α-LP demonstrated the ability to bind to the tumor neovasculature, inducing endothelial cell lysis and blood flow disruption through neutrophil recruitment.^306^ The subsequent inflammatory response and release of proinflammatory cytokines, such as TNF-α and IFN-γ, further amplify the destruction of the tumor vasculature and contribute to TME remodeling.^298^ Moreover, some OVs, such as VV and HSV, have been engineered to express antiangiogenic proteins, including angiostatin and endostatin, which inhibit angiogenesis by inducing apoptosis in proliferating endothelial cells and suppressing VEGF signaling.^284,307^ For example, preclinical studies revealed that endostatin-armed HSV enhanced vascular collapse and improved tumor control.^308^

Tumor angiogenesis is regulated by several factors, including oncogene-driven protein expression, cellular stress (e.g., hypoxia, low pH, and nutrient deprivation), and aberrant signaling pathways, such as the VEGF/EGFR/Ras pathways.^309,310^ These factors not only support tumor vascularization but also increase the susceptibility of the vasculature to OV infection and replication. Dysregulated VEGF/EGFR signaling in the tumor-associated vasculature, for example, facilitates VV infection, resulting in vascular leakage and collapse.^305^ Furthermore, VEGF signaling suppresses the antiviral immune response by activating the MAPK and STAT3 pathways and upregulating PRD1-BF1/Blimp1 expression in the tumor vasculature, enabling OVs to replicate in endothelial cells.^301^ Following infection, OVs downregulate VEGF expression, further inhibiting angiogenesis.^311^ Equipping OVs with antiangiogenic genes represents a promising strategy to enhance these effects. For example, the VEGF-armed OAd has been shown to inhibit neovascularization by inducing apoptosis in proliferating endothelial cells.^312^ Similarly, VV and OAd systems armed with α-VEGF antibodies have been developed, resulting in decreased microvessel density and improved tumor control.^301,313^ These strategies increase vascular collapse while promoting systemic immune infiltration into the TME, thereby synergizing with immunotherapies such as ICIs.

## OVs combined with other therapies

**Fig. 4:**
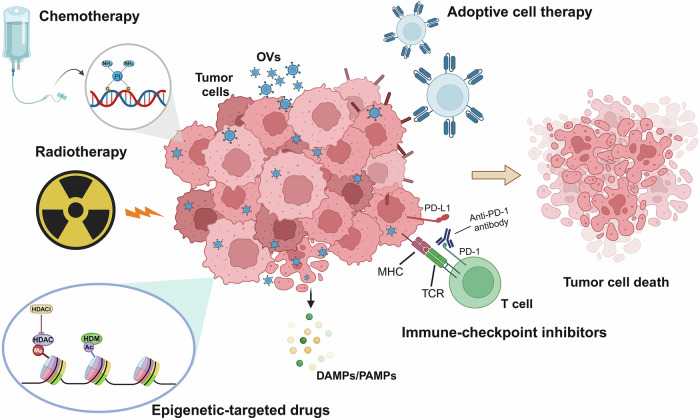
Potential OV combination strategies in clinical development. OVs, when combined with traditional therapies such as chemotherapy and radiotherapy, induce tumor cell lysis, releasing TAAs, viral antigens, and DAMPs. These released molecules activate immune responses, helping to overcome immune evasion. OVs can also enhance the effectiveness of ICIs by overexpressing immune checkpoint molecules such as PD-L1 on tumor cells, transitioning the TME from a “cold” state (low immune cell infiltration) to a “hot” state (high immune cell infiltration), thus promoting antitumor immunity. Furthermore, the combination of OVs with adoptive cell therapy leads to enhanced T-cell-mediated tumor destruction by facilitating T-cell penetration into the tumor and increasing cytokine production within the TME. Additionally, epigenetic-targeted drugs can enhance the ability of the immune system to recognize and eliminate tumor cells, improving the overall efficacy of OV therapy. TAAs tumor-associated antigens, PAMPs pathogen-associated molecular patterns, DAMPs damage-associated molecular patterns. Created with BioRender.com

Despite the substantial antitumor activity observed in numerous preclinical and clinical studies, indicating its great potential as a novel immunotherapy approach, the efficacy of OVs as monotherapies remains limited, akin to many conventional cancer treatments.^11^ This limitation is attributed primarily to resistance mechanisms driven by tumor heterogeneity, complex genetic mutations, and the intricate composition of the TME. These factors pose significant challenges for single-agent therapies, including OVs, in achieving optimal antitumor effects independently.^314^ However, OVs exhibit considerable flexibility, enabling them to deliver key immunomodulatory factors directly into the TME, remodeling the TME and inducing a robust immune response. Consequently, OV-based combination therapies have emerged as highly promising strategies, substantially increasing antitumor efficacy. Moreover, the mechanisms by which OVs exert their effects differ fundamentally from those of other anticancer therapies.^315,316^ The unique toxicity profiles of OVs generally do not overlap with those of other treatments, allowing OVs to synergize with diverse therapeutic modalities and mitigate adverse effects. These properties make OVs ideal candidates for inducing personalized immune responses and integrating seamlessly with a wide range of other treatments, including chemotherapy, radiotherapy, and immunotherapies such as ICIs, CAR-T-cell therapy, adoptive T-cell therapy and epigenetic targeted drugs (Fig. 4). In conclusion, combining OVs with other therapeutic approaches not only maximizes their potential and enhances their antitumor effects but also addresses the limitations of monotherapy. This integrative and effective strategy for cancer treatment improves therapeutic outcomes and provides renewed hope for patients.^317^

### OVs and ICIs

ICIs are evolutionarily conserved immune regulators essential for maintaining immune homeostasis. However, tumor cells exploit these molecules to evade immune surveillance, enabling tumor progression despite an otherwise functional immune system.^318,319^ Immune checkpoint molecules, such as cytotoxic T lymphocyte antigen 4 (CTLA-4), lymphocyte activation gene 3 (LAG-3), T-cell immunoglobulin and mucin-domain containing-3 (TIM-3), and T-cell immunoreceptor with Ig and ITIM domains (TIGIT), regulate T-cell activation and promote immunosuppression within the TME. In particular, the PD-1/PD-L1 axis operates during the effector phase of T-cell responses, diminishing cytotoxic capacity and facilitating immune evasion.^320^ This interaction creates an immunosuppressive TME, promoting cancer progression despite a functional immune system. Consequently, ICIs disrupt these inhibitory interactions, restoring immune-mediated tumor eradication.^321^ For example, anti-CTLA-4 (e.g., ipilimumab), anti-PD-1 (e.g., nivolumab), and anti-PD-L1 (e.g., atezolizumab) agents have demonstrated durable responses in several cancers, including melanoma, NSCLC and renal cell carcinoma.^322–324^ However, the efficacy of ICIs is often constrained by both intrinsic and extrinsic factors within the TME. These factors include low levels of TILs, reduced expression of TAAs or tumor-specific antigens (TSAs), and an abundance of immunosuppressive cells, such as Tregs and MDSCs.^325^ Tumors exhibiting an immunologically “cold” phenotype, characterized by low immunogenicity and minimal immune cell infiltration, are particularly resistant to ICIs.^326^ Thus, innovative strategies aimed at modulating the TME and enhancing immune cell infiltration are essential for optimizing ICI efficacy.

OVs have emerged as promising agents for enhancing TME immunogenicity and overcoming resistance to ICIs. OVs selectively replicate within tumor cells, causing direct oncolysis and the release of TAAs, TSAs, and DAMPs. This process induces a robust inflammatory response characterized by the production of type I IFNs, proinflammatory cytokines (e.g., TNF-α and IL-6), and chemokines (e.g., CCL5 and CXCL10), which recruit TILs into the TME.^327,328^ Moreover, OVs facilitate the release of soluble tumor antigens and enhance antigen presentation by DCs, leading to the activation of tumor-specific T cells. This process increases T-cell infiltration and primes the immune system for more effective ICI therapy.^329,330^ Interestingly, OVs can upregulate the expression of immune checkpoint molecules such as PD-L1 and CTLA-4 on tumor cells, increasing tumor susceptibility to ICIs. This complementary relationship highlights the potential synergy between OVs and ICIs in antitumor therapy.^331,332^ Additionally, local intratumoral administration of OVs can induce an abscopal effect, wherein noninjected tumors regress due to systemic immune activation, thereby increasing ICI efficacy and reducing the risk of systemic toxicity.^134,333–335^

The combination of local OVT with systemic ICI therapy has shown efficacy in several trials. For example, in a phase Ib melanoma trial (NCT02263508), the combination of T-VEC with pembrolizumab (anti-PD-1) achieved an overall objective response rate (ORR) of 62% in patients with unresectable, previously untreated stage IIB to IV melanoma, with no DLTs.^336,337^ This outcome significantly surpasses historical response rates for either treatment alone, supporting the hypothesis that OVs can enhance the immune response induced by ICIs and improve clinical efficacy, particularly in tumors with high immunogenicity or those that are more responsive to immune modulation. Similarly, a phase II trial (NCT01740297) combining T-VEC with ipilimumab (anti-CTLA-4) in 198 patients with stage IIIB to IV melanoma demonstrated a significantly improved ORR compared with ipilimumab alone.^338^ Furthermore, OV-ICI combinations have proven effective in advanced sarcoma, with 30% of patients achieving optimal responses despite being PD-L1-negative at baseline.^339^ These findings demonstrate that OV-ICI combinations may improve outcomes even in challenging tumor types, such as sarcoma, which is notorious for its limited treatment options. Additionally, OVs can serve as neoadjuvant agents, increasing the efficacy of tumor resection and subsequent ICI therapy. For example, early administration of oncolytic Maraba virus and reovirus, followed by surgical resection and PD-1 inhibitor treatment, has led to increased cytotoxic T-cell infiltration and prolonged survival in triple-negative breast cancer (TNBC) models and glioma patients.^148,340,341^ These findings underscore the potential of OV-ICI combinations to convert immunologically “cold” tumors into “hot” tumors characterized by robust immune cell infiltration and activation.

Despite these encouraging results, OV-ICI combinations have not always demonstrated the anticipated synergy in clinical settings. Compared with pembrolizumab alone, the MASTERKEY-265 phase III trial (NCT02263508), which evaluated the combination of T-VEC with pembrolizumab in advanced melanoma, failed to significantly improve PFS (HR, 0.86; P = 0.13) or OS (HR, 0.96; P = 0.74).^342^ This discrepancy underscores the need to further investigate the factors influencing the efficacy of OV-ICI combinations. One possible explanation for this discrepancy is the inherent heterogeneity of tumors, especially in advanced stages, where the TME is more immunosuppressive. Advanced melanoma often employs immune escape mechanisms, including elevated levels of Tregs and MDSCs, which can overwhelm the immunostimulatory effects of OVT. These immunosuppressive cells contribute to a “cold” TME that limits both OV-induced immune priming and the effectiveness of ICIs. Moreover, the sequence and timing of treatment may also play a critical role in determining therapeutic success. Preclinical studies indicate that initial OV treatment can prime the immune system by inducing an inflammatory TME, thereby enhancing subsequent ICI efficacy. However, if ICIs are administered at the wrong time, either too early or concurrently, immune priming may not reach its full potential, diminishing the synergistic effects of the combined therapy. Furthermore, patient-specific factors, such as immune status, genetic background, and tumor characteristics, likely contribute to the heterogeneous observed responses to OV-ICI combinations. Tumors with high PD-L1 expression or a substantial mutational burden may respond better to these therapies, whereas those with low immunogenicity or enhanced immune evasion mechanisms may benefit less. Collectively, these factors, including tumor heterogeneity, TME immunosuppression, and patient-specific variations, highlight the complexity of OV-ICI combination therapy and emphasize the need for personalized treatment strategies tailored to individual tumor profiles and immune landscapes.

The efficacy of OV-ICI combinations is influenced by multiple factors, including OV type, ICI selection, administration sequence, and patient-specific characteristics. Preclinical studies highlight the importance of optimizing treatment sequencing and timing.^343,344^ Available administration strategies include (i) OV-primed ICI therapy, (ii) concurrent therapy, (iii) ICI-primed OV therapy, (iv) OV-primed concurrent therapy, (v) concurrent therapy with armed OVs, and (vi) alternating OV and ICI administration.^343^ Although no definitive consensus has been reached regarding the optimal sequencing strategy, a promising approach involves initiating therapy with an OV lead-in, followed by synchronous ICI administration. This sequence facilitates OV-induced immune cell infiltration, potentially creating an immunologically “hot” TME. However, this effect may be counteracted by the upregulation of PD-L1 on tumor cells. Thus, simultaneous administration of OV and ICI after initial OV treatment is essential to sustain the inflammatory TME and prevent PD-L1-mediated T-cell exhaustion.

Other factors, including cancer type, viral strain, immune response dynamics, and treatment duration, further influence the efficacy of OV-ICI combinations. A patient-tailored approach that integrates genomic profiling and TME characterization may enhance the therapeutic efficacy of this combination therapy. For example, tumors with high PD-L1 expression may benefit from OVs that induce further PD-L1 expression, thereby increasing the efficacy of ICIs.^331^ Additionally, engineering OVs to express immune checkpoint antibodies or cytokines (e.g., GM-CSF and IL-12) within the TME offers a promising approach for targeted immune modulation while minimizing systemic toxicity. While the combination of OVs and ICIs has significant antitumor potential, further studies are needed to refine treatment sequencing, optimize administration timing, and develop patient-specific strategies that enhance efficacy and clinical outcomes.

### OVs and ACT

Adoptive cell therapy (ACT) involves transferring cultured lymphocytes with antitumor activity into patients to induce tumor regression. ACTs include CAR-T-cell therapy, CAR-NK-cell therapy, engineered T-cell receptor (TCR-T)-cell therapy, and TIL therapy.^345–347^ Despite the remarkable success of ACT in hematologic malignancies and melanoma, its efficacy in solid tumors remains limited due to several intrinsic challenges, including poor engraftment and persistence of transferred cells, inefficient tumor infiltration, suboptimal target recognition, and an immunosuppressive TME.^348,349^ The combination of OVs with ACTs has emerged as a promising strategy to overcome these obstacles by leveraging the complementary strengths of both modalities.

Efficient trafficking of CAR-T and TCR-T cells to the tumor core is essential for effective tumor eradication. However, this process is impaired by aberrant chemotactic signaling, disorganized tumor vasculature, and the presence of immunosuppressive cells, including Tregs and tumor-associated macrophages (TAMs).^350^ OVs can improve T-cell migration by modulating the TME and promoting positive chemokine–chemokine receptor interactions. For example, OVs engineered to express chemokines such as CCL5 and CXCL11 effectively recruit DCs, CD8^+^ cytotoxic T cells, and CD4^+^ helper T cells to the tumor site.^267,351^ Moon et al. engineered CAR-T cells to express CXCL11 (CAR/CXCL11) and paired them with an oncolytic VV (VV.CXCL11) in a preclinical study.^268^ While both CAR/CXCL11 and VV. CXCL11 increased CXCL11 protein levels within tumors, but only VV.CXCL11 effectively recruited T cells and significantly enhanced antitumor efficacy, demonstrating the superiority of OVs as partners for CAR-T-cell therapy. Similarly, OAd expressing CCL5 increased CAR-T-cell infiltration into solid tumors, improving therapeutic outcomes.^352^

T cells often experience exhaustion and exhibit diminished persistence within the suppressive TME.^353^ Cytokine delivery is crucial for sustaining T-cell survival and expansion. While CAR-T cells and TCR-T cells can be modified to deliver cytokines such as IL-12 and IL-15, OVs exhibit a superior capacity for localized intratumoral cytokine delivery. OVs can reprogram the TME, facilitating ACT trafficking to the tumor site while supporting their survival and expansion, thereby mitigating the exhaustion of adoptively transferred cells and creating a more favorable environment for ACT infiltration and activation.^354^ For example, in a study in which an IL-7-loaded OAd was combined with B7H3-targeted CAR-T cells, the treatment significantly improved T-cell proliferation, reduced apoptosis, and enhanced the therapeutic efficacy against glioblastoma.^355,356^ IL-21-armed OVVs synergized with CAR-T-cell therapy to increase TIL activity, reduce the proportion of exhausted PD-1^hi^Tim-3^+^ T cells and Tregs, and improve tumor responses.^357^ These studies demonstrate that cytokine-armed OVs not only improve the persistence of ACT cells but also modulate the TME to favor antitumor immunity.

A major limitation of CAR-T and TCR-T-cell therapies in solid tumors is the loss or heterogeneity of target antigens.^358^ To address this challenge, OVs have been employed as carriers for bispecific T-cell engagers (BiTEs). BiTEs enable T cells to recognize and attack tumor cells expressing diverse antigens, thus overcoming antigen escape. For example, OAd-mediated delivery of an EGFR-targeting BiTE (OAd-BiTE) improved the efficacy of folate receptor alpha (FR-α)-specific CAR-T-cell therapy by redirecting T cells to EGFR-positive, FR-α-negative cancer cells. This approach reduces tumor heterogeneity and extends survival in preclinical models.^359^ Similarly, a trivalent OV (CAdTrio), engineered to produce IL-12, an anti-PD-L1 antibody, and CD44 variant 6 (CD44v6)-targeting BiTEs, enabled dual targeting of HER2^+^ and HER2^−/−^ CD44v6^+^ tumors when combined with HER2-specific CAR-T cells.^360^ These results highlight the ability of BiTE-armed OVs to broaden the antigenic scope and enhance the antitumor efficacy of ACT.

Solid tumors exhibit an immunological “cold” phenotype characterized by a lack of infiltrating CD8^+^ T cells, the presence of immunosuppressive cells such as Tregs and MDSCs, and reduced expression of MHC-I and immunostimulatory markers such as PD-L1. As a result, ACT cells struggle to effectively penetrate the tumor core and sustain cytotoxic activity within the suppressive TME.^361^ To overcome these barriers, the TME must be modified to support an inflammatory, “hot” phenotype that enhances ACT efficacy.^362^ Likewise, OAd Delta-24-RGDOX combined with ovalbumin-specific T cells demonstrated robust intratumoral immune activation and systemic antitumor immunity, effectively inhibiting distant metastases.^356^

T-cell exhaustion and senescence are major obstacles in ACT and are characterized by the upregulation of inhibitory receptors such as PD-1, CTLA-4, and TIM-3. Elevated PD-1 expression in TCR-T cells after infusion has been associated with reduced IFN-γ production and a diminished immune response.^363^ T-cell exhaustion impairs the functional capacity of infused T cells and accelerates their attrition within the hostile TME. Disruption of the PD-1/PD-L1 axis is a promising strategy to alleviate T-cell exhaustion and improve the persistence of adoptively transferred T cells.^364^ One strategy to augment T-cell function and persistence in tumors involves the self-delivery of PD-1 blocking agents via engineered CAR-T cells.^365^ This strategy involves genetically modifying T cells to express a small fragment of an anti-PD-1 antibody, such as a single-chain variable fragment (ScFv), to locally disrupt the PD-1/PD-L1 interaction in the TME without systemic administration. Furthermore, armed OVs offer a potentially safer strategy to further increase ACT efficacy. The localized secretion of functional checkpoint blockade factors by OVs has demonstrated potential in sustaining T-cell function and persistence at tumor sites. For example, compared with systemic PD-1 blockade, an OAd (CAd-VECPDL1) expressing an anti-PD-L1 mini-antibody improved the antitumor activity of HER2-specific CAR-T cells in prostate cancer models.^366^ The combination of CAd-VECPDL1 with HER2-targeted CAR-T cells amplifies the effects of CAR-T and TCR-T cells by maintaining T-cell functionality and mitigating immune suppression within the TME.

Adoptively transferred immune cells can also serve as systemic carriers to deliver OVs to tumor sites. For example, Zheng et al. employed CAR-T and TCR-T cells as efficient vectors to deliver myxoma virus to antigen-expressing tumors, inducing tumor-specific cell death, autophagy, and bystander killing effects. This approach contributes to the eradication of antigen-negative tumor cells, the establishment of adaptive immunity, and the inhibition of antigen escape.^367^

Despite the substantial benefits of combining OVs with CAR-T cells, determining the optimal timing and sequence of administration remains crucial.^368^ Studies have shown that coadministration of OVs and CAR-T cells results in limited tumor reduction, whereas sequential administration yields better results. The scheduling and dosage of OVs and CAR-T cells are key determinants of treatment outcomes. Numerous unresolved factors, such as optimal dosage ratios, administration schedules, and genetic engineering strategies, require further investigation to maximize synergy in combination therapies. Additionally, CAR-T-cell therapy induces adverse effects such as cytokine release syndrome (CRS) and neurotoxicity. Therefore, safety concerns must be carefully evaluated when integrating CAR-T-cell therapy with OVs.^369^

Compared with T cells, NK cells possess inherent immunotherapeutic advantages, such as not requiring human leukocyte antigen (HLA) matching and not causing graft-versus-host disease (GVHD).^370^ Moreover, unlike autologous or HLA-matched adoptive T-cell therapy, NK-cell therapy can utilize unmatched allogeneic NK cells from a single donor batch to treat multiple patients with the same cancer type. The “off-the-shelf” availability of NK cells enhances their potential for large-scale clinical applications and commercialization.^371^ However, the use of NK cells or CAR-NK cells in combination with OVT remains in the early investigational stage. In a colon cancer model, CCR5-overexpressing NK cells combined with CCL5-modified VV demonstrated superior efficacy compared with either agent alone, with significantly greater NK cell infiltration in the TME than that seen with the prototype virus.^372^ In multiple glioblastoma mouse models, HSV-1-based OVs expressing the IL15/IL15Rα complex (OV-IL15C), when combined with frozen EGFR-CAR-NK cells, enhanced NK and CD8^+^ T-cell infiltration and activation, thereby extending CAR-NK cell persistence and inhibiting tumor growth.^373^ In a phase I/II trial (NCT03056339), 11 patients with relapsed or refractory CD19-positive cancers received cord blood-derived CAR-NK cell therapy, which resulted in sustained cell expansion and persistence over 12 months without neurotoxicity, CRS, or GVHD.^374^

In summary, combining OVs with ACT provides a novel strategy for treating solid tumors by optimizing the TME and enhancing both the infiltration and functional activity of adoptive cell therapies. This combination offers new avenues to overcome the limitations of traditional ACT in treating solid tumors. Future research should explore optimal OV‒ACT combination models to achieve broader clinical applications and improved treatment outcomes.

### OVs and radiotherapy

Radiotherapy remains a fundamental treatment for localized tumors, leveraging ionizing radiation to induce direct DNA damage in cancer cells or generate ROS that disrupt cellular processes.^375^ Although radiotherapy has proven effective in local tumor control, its effectiveness is often limited by tumor resistance and off-target toxicity to healthy tissues.^376^ Emerging evidence indicates that combining radiotherapy with OVT can significantly enhance therapeutic outcomes by addressing the limitations of each modality. The synergistic effect between radiotherapy and OVs offers a promising strategy for treating aggressive and resistant malignancies.

The combination of OVs and radiotherapy elicits notable synergistic antitumor effects, especially in aggressive malignancies resistant to conventional treatments. While the exact mechanisms underlying this synergy remain to be elucidated, several hypotheses have been proposed: (1) Increased radiosensitivity: OVs enhance tumor radiosensitivity by interfering with DNA damage repair pathways.^377,378^ For example, certain OVs sequester DNA repair proteins such as RAD51, which amplifies radiation-induced DNA damage and cytotoxicity.^379^ This radiosensitization effect is tumor specific, minimizing collateral damage to healthy tissues. Preclinical studies involving OAd Delta-24-RGD in glioblastoma models have shown that downregulating DNA damage repair proteins enhances radiotherapy efficacy and extends survival.^35^ (2) Improved viral uptake and replication: RT-induced apoptosis releases TAAs and DAMPs, fostering a proinflammatory environment that facilitates OV replication and dissemination.^380^ Additionally, radiotherapy increases the permeability of the tumor vasculature, facilitating OV infiltration and intratumoral replication.^381^ For example, radiation upregulates the expression of Dynamin 2, which leads to Ad internalization upon binding to the CAR or integrins, enhancing adenoviral entry into tumor cells.^382^ A TRAIL-expressing OAd combined with radiotherapy in colorectal cancer models resulted in dose-dependent tumor regression, likely due to enhanced viral spread and oncolysis.^383^ (3) Enhanced abscopal effect: The abscopal effect refers to the regression of nonirradiated tumors due to systemic immune activation triggered by local radiotherapy.^384^ OVs enhance this effect by promoting antigen presentation and immune cell recruitment, thereby broadening systemic immune responses.^385^ In a study involving NDV and radiotherapy, combination therapy not only improved local tumor control but also delayed progression in distant, untreated tumors, highlighting the potential for systemic antitumor benefits.^386^ (4) Immune system activation: Radiotherapy-induced cell death releases TAAs and DAMPs, such as calreticulin, ATP, and HMGB1, which prime DCs and activate tumor-specific T cells. This process synergizes with the immunostimulatory effects of OVs, further augmenting the immune response against tumors.^385^ In prostate cancer xenograft models, combining Ad5/3-D24-hTNFα with radiotherapy resulted in significant tumor regression, possibly attributed to the increased release of ATP, calreticulin, and HMGB1, key factors of ICD.^387^ The integration of DNX-2401 and radiotherapy in glioblastoma models increases CD3^+^ and CD8^+^ T-cell infiltration, improving antitumor immunity.^388^ (5) Disruption of the tumor vasculature: RT-induced vascular damage increases tumor permeability, facilitating OV spread throughout the tumor mass. This vascular disruption enhances the distribution and therapeutic efficacy of OVs, particularly in poorly vascularized tumors.

Clinical trials are exploring the safety and efficacy of combining radiotherapy with OVs and have shown promising preliminary outcomes across multiple cancer types. A phase I trial investigated OBP-301, an OAd that targets the TERT promoter, combined with radiotherapy in esophageal cancer patients. This combination was well tolerated and demonstrated promising therapeutic benefits, including tumor stabilization and regression, particularly in patients who were intolerant to standard therapies.^389^ In pediatric patients with high-grade glioma and diffuse intrinsic pontine glioma (DIPG), a single intratumoral injection of Delta-24-RGD followed by radiotherapy improved T-cell activation and reduced the tumor burden in 9 out of 12 patients, underscoring the potential of integrating OV-based immunotherapy into standard radiotherapy protocols.^388^ Another study by Freytag et al. reported a 42% reduction in biopsy positivity of prostate cancer in patients receiving combined OV and radiotherapy treatment two years postintervention.^390^ Moreover, triple combination therapy involving cisplatin, radiation, and intravenous delivery of oncolytic VV (GL-ONC1) was found to be safe and feasible in a phase I trial in patients with head and neck cancer.^391^ Recent studies have highlighted the potential of OVs as radiosensitizers, especially DNA-based OVs capable of replicating within host cell nuclei. Numerous studies have explored the broad-spectrum antitumor effects of radionuclide therapy combined with oncolytic VSV, HSV, MV, and other viruses genetically modified to express NIS, which drives the cellular uptake of radionuclides, such as ^131^I.^171,392,393^

### OVs and chemotherapy

Traditional cytotoxic agents, such as DNA intercalators, nucleotide analogs, and alkylating agents, primarily target rapidly dividing cells by inhibiting DNA synthesis and disrupting mitosis.^50^ However, chemotherapy is often limited by systemic toxicity, resistance development, and collateral damage to normal tissues, and innovative strategies to improve both efficacy and specificity are needed.^394,395^ In contrast, OVs exhibit high tumor specificity due to their natural tropism and genetic modifications, which minimize off-target effects and enhance safety profiles.^377^ The distinct mechanisms of action associated with chemotherapy and OVs provide a strong rationale for their combination, potentially producing synergistic antitumor effects.^7^

The synergy between chemotherapy and OVs is driven by several complementary mechanisms. OVs and chemotherapeutic agents can cooperatively increase apoptosis in tumor cells. Chemotherapeutic agents frequently induce ICD, releasing DAMPs and soluble tumor antigens. These molecules enhance OV-mediated immune activation by expanding neoantigen repertoires and stimulating tumor-specific T-cell responses.^132 Th^is immune activation enhances the clearance of cancer cells and reduces the likelihood of tumor recurrence. For example, gemcitabine combined with an OAd engineered to express relaxin not only induced apoptosis in a pancreatic xenograft model but also degraded the ECM, which often serves as a barrier to OV spread and contributes to chemotherapy resistance.^396^ This approach significantly enhanced tumor control. Similarly, the combination of oncolytic HSV-1 with mitoxantrone has been shown to increase antigen-specific CD8^+^ T-cell infiltration in tumors, augmenting apoptosis and promoting a robust antitumor immune response.^397^ Chemotherapeutic drugs such as cyclophosphamide have been shown to modulate the TME by depleting Tregs and other immunosuppressive components.^398^ This modulation creates a more favorable immune environment for OVs, transforming “cold” tumors into “hot” tumors with increased immune infiltration. For example, low-dose cyclophosphamide enhances OV replication and antitumor activity by alleviating immunosuppression within the TME.

Compared with monotherapy, combining OVs with chemotherapeutic agents significantly enhances therapeutic efficacy. When ONYX-015 is combined with cisplatin and 5-fluorouracil (5-FU), the objective response rate reaches 65%, significantly exceeding the 15% response rate observed with ONYX-015 monotherapy.^399^ Combining vincristine with OAd SG600 inhibited tumor growth by modulating the cell cycle and reducing protein kinase B phosphorylation, a key factor in chemotherapy resistance, thereby increasing tumor cell sensitivity to vincristine. Further studies confirmed that vincristine does not hinder the replication of OAd SG600, ensuring effective oncolysis.^400^

The optimal sequencing of OV and chemotherapeutic agent administration remains controversial. Immune cells activated by OVs may be targeted by chemotherapeutic agents, which can also exert antiviral effects, reducing viral replication in the TME and diminishing the synergy of combination therapy. Preclinical studies suggest that cyclophosphamide pretreatment enhances OV efficacy, indicating that chemotherapy pretreatment might optimize combination treatment outcomes. Although clinical studies remain limited, emerging data highlight the promise of combining OVs with cytotoxic agents. For example, a phase I/II trial evaluating the combination of carboplatin and paclitaxel with reovirus in patients with head and neck cancer demonstrated synergistic cytotoxic activity and a favorable objective response, with minimal antiviral immunity.^401^ Optimizing the sequence and combination of these treatments could enhance both the efficacy and safety of OV-chemotherapy regimens. Administering OVs prior to chemotherapy to activate the immune system may increase tumor cell lysis and amplify the overall immune response, ultimately reducing the tumor burden.^402^ This strategy may enable OVs to prime the immune system, increasing the susceptibility of tumor cells to subsequent chemotherapy-induced cytotoxicity. Conversely, chemotherapy administered first to reduce the tumor burden and modulate the TME may enhance OV replication and oncolytic efficacy. This approach leverages the capacity of chemotherapy to decrease the tumor burden and alter the immune landscape, thereby improving OV replication and efficacy. The pharmacodynamics underlying this combination strategy have been further explored in recent studies. For example, a study involving VG161 in combination with gemcitabine and nab-paclitaxel in a murine pancreatic cancer model reported the most favorable outcomes when VG161 was administered postchemotherapy. In contrast, the concurrent administration of both treatments provides no discernible advantage over monotherapy. This finding suggests that, in certain cases, chemotherapy may create an optimal posttreatment environment for OV replication, which is not achieved when both treatments are administered simultaneously. Similarly, previous studies examining the combination of ONYX-015 with cisplatin in head and neck squamous cell carcinoma xenograft models have shown that the sequence of administration influences survival outcomes, with OV application after chemotherapy yielding superior results compared with concurrent therapy.^403^ The intricate interplay between these therapies warrants further investigation via advanced techniques, such as single-cell RNA sequencing and high-throughput methodologies. These approaches provide a deeper understanding of chemotherapy–OV interactions at the molecular level, facilitating biomarker identification for improved clinical decision-making. By investigating the cellular and molecular mechanisms governing the efficacy of these combination therapies, researchers can develop strategies to maximize their synergistic potential while minimizing adverse effects.^402,404^

### OVs and epigenetic targeted drugs

Epigenetic modifications, such as DNA methylation, histone modifications, and chromatin remodeling, play crucial roles in cancer initiation, progression, and therapy resistance.^405^ These modifications often result in tumor suppressor gene silencing and oncogene activation, fostering a permissive TME that supports cancer progression.^406,407^ Epigenetic-targeting drugs, including histone deacetylase inhibitors (HDACis), DNA methyltransferase inhibitors (DNMTis), and bromodomain and extraterminal domain inhibitors (BETis), aim to reverse these aberrant changes.^408^ When integrated with OVs that selectively infect and kill tumor cells, this combination offers a novel and synergistic therapeutic strategy for cancer treatment.

Epigenetic inhibitors, particularly HDACis, have been shown to increase OV replication within tumor cells.^409,410^ For example, HDACis such as vorinostat and MS-275 have been reported to enhance the oncolytic activity of viruses such as VSV (VSVΔ51) and Ad.^411,412^ This synergy arises from the inhibition of interferon-stimulated genes (ISGs) and the subsequent suppression of antiviral responses, which typically hinder virus replication and dissemination within the tumor.^409,413,414^ Suppressing ISGs allows the virus to replicate more effectively, leading to enhanced oncolysis and tumor apoptosis. Epigenetic drugs can upregulate cell surface receptors essential for OV entry. For example, DNMTis have been shown to upregulate coxsackievirus and adenovirus receptors, increasing OAd infectivity in cancer cells.^412,415,416^ Similarly, HDACis can increase nectin receptor expression, improving the efficacy of HSV-based OVs.^417^ Epigenetic drugs can also modulate the TME to enhance immune responses.^418^ For example, HDACis have been shown to induce the expression of NK cell-activating ligands and TAAs, which boosts the priming of NK cells and CTLs.^419^ This immune activation enhances systemic antitumor immunity triggered by OVs, providing a dual mechanism of action: direct viral oncolysis and immune-mediated tumor destruction. In addition, HDACis have been reported to enhance the transcriptional activity of virally encoded transgenes, amplifying tumor-specific cell death.^420^ A major challenge in cancer therapy is the development of resistance to standard treatments, including chemotherapy and radiotherapy. The combination of OVs with epigenetic drugs represents a novel strategy to overcome this resistance.^421^ For example, HDACis have been shown to sensitize tumor cells to OVs by modulating the expression of genes associated with stress responses and apoptosis. In particular, the combination of HDACis with OVs enhances tumor cell susceptibility to oncolysis by decreasing the number of cellular resistance mechanisms typically induced by chemotherapy or radiotherapy.^422,423^

Multiple preclinical studies have demonstrated the synergistic effects of combining OVs with epigenetic therapies. For example, in prostate cancer models, combining VSVΔ51 with vorinostat significantly enhanced viral replication and promoted tumor cell death.^423^ Similarly, vorinostat combined with an HSV-1-based OV increased viral replication and tumor regression in glioblastoma models.^411^ Additionally, HDACis enhanced CD8^+^ T-cell recruitment, amplifying antitumor immune responses. Although preclinical data are robust, clinical trials investigating OV-epigenetic drug combinations remain in the early stages. A phase I trial combining the DNMTi decitabine with a reovirus-based OV demonstrated safety and preliminary efficacy in advanced solid tumors, supporting further investigations.^424^

The integration of OVs with epigenetic-targeted therapies offers several advantages, including enhanced viral replication, an improved immune response, and reduced tumor resistance. This therapeutic strategy provides a more precise approach to tumor eradication by utilizing both direct oncolytic activity and immune system modulation.^425^ However, challenges persist, including determining the optimal sequencing of treatments and managing potential off-target effects of epigenetic modulators. While epigenetic therapies can prime tumors for OV infection, excessive immune activation may diminish OV efficacy. Determining the optimal timing and dosage of these combinations requires further investigation.^412^ Broad-spectrum epigenetic drugs may have off-target effects on normal cells. The development of tumor-specific epigenetic modulators could increase therapeutic precision. Finally, despite promising preclinical results, translating OV-epigenetic drug combinations into clinical practice requires extensive trials to assess their safety, efficacy, and long-term benefits.

## Clinical challenges of OVT

### Delivery challenge

The clinical efficacy of OVs is frequently constrained by delivery challenges. Effective viral delivery methods are crucial to ensure that OVs reach the tumor site, elicit therapeutic effects, and spare normal tissues. Currently, the two main delivery routes used in clinical trials are intratumoral injection (i.t.) and intravenous infusion (i.v.).^426^ Intratumoral injection delivers viral particles directly to the tumor site, offering high safety and reducing the risk of circulating antibodies clearing the virus before it reaches the tumor.^427–429^ However, intratumoral injection carries risks such as bleeding and inadvertent metastasis, making it unsuitable for widespread or difficult-to-reach tumors.^430^ This limitation is also observed with T-VEC, an approved therapy restricted to intratumoral administration.^431 In^ contrast, intravenous delivery can target multiple lesions simultaneously, making it particularly suitable for tumors that are physically inaccessible.^259,432–434^ It also eliminates the need for precise localization techniques, as it is relatively noninvasive and highly repeatable. However, intravenous injection may cause premature clearance of viral particles, reducing therapeutic efficacy. Additionally, once diluted in the peripheral circulation, predicting the bioavailable concentration at the tumor site becomes challenging.^435^ Intraperitoneal, intrathecal, intracranial, and intrapleural injections are commonly used for targeting intra-abdominal organs, the central nervous system, and lung tumors, respectively.^436^ However, these routes are mainly used in laboratory animals and have limited clinical applicability. The optimal route of administration remains debated, with no universally established guidelines. Less invasive routes, such as oral/mucosal and nasal administration, appear to improve patient acceptability, especially for gastrointestinal and cerebral malignancies. These routes should be considered in future studies. To enhance the distribution efficiency and therapeutic effect of OVs, further research on intravenous delivery is necessary to better understand the pharmacokinetics of specific OVs in systemic circulation.

The development of innovative delivery systems offers potential solutions for overcoming these limitations. For example, cell-based or bionanocarrier delivery strategies aim to increase viral targeting and bioactivity. Cell-based strategies, such as those utilizing bone marrow-derived mesenchymal stem cells, have shown effectiveness. Stem cells, including mesenchymal and neural stem cells, are considered ideal drug vehicles because of their natural tumor-homing properties and low immunogenicity.^437,438^ Immune cells are also employed for OV delivery.^439^ Previous reports have suggested that macrophages can effectively migrate to hypoxic regions within tumors. However, these strategies are generally applied to specific virus types, and the proliferative capacity and activity of carrier cells decrease over time, limiting their long-term effectiveness in tumor treatment.^440^ To address these challenges, Chen et al. developed the ONCOTECH delivery system, which physically attaches membrane-coated OVs to the surface of T cells via TCRs or CARs. This approach not only protects OVs from neutralization but also enhances their tumor targeting ability, ensuring synchronized pharmacokinetics for both cells and viruses. However, ONCOTECH faces challenges in treating late-stage solid tumors with antigen loss or defects.^441^ Bionanomaterial-based carriers also encounter issues such as limited clinical applicability, nonspecific uptake, and rapid clearance.^442^ However, some innovative bionanomaterial carriers, such as bioengineered cell membrane nanovesicles designed by Peng et al.,^443^ have demonstrated longer circulation times, higher survival rates, and enhanced efficacy of encapsulated OAds.^444^

In summary, effective OV delivery continues to face numerous challenges. However, with continuous technological improvements and strategy optimization, significant enhancements in clinical efficacy are anticipated.

### Safety concerns

Although OVs generally have an acceptable safety profile, their use as live replicating organisms necessitates special precautions, including the risks of viral shedding and unintentional transmission to healthcare workers, close contacts, and the environment. To minimize these risks, comprehensive safety guidelines for the storage, handling, and administration of OVs must be established, along with protocols for managing accidental spills, overdosing, and disinfecting areas that may come into contact with the virus.^445–447^ Additionally, specific measures must be developed to address the potential for viral exposure and accidental transmission through needles, wounds, or contaminated materials.^448^ The safe use of OVs also depends on educating healthcare professionals and providing patients with guidance on managing injection sites.

Viral shedding, a well-documented phenomenon in OVT, presents another safety concern. Although viral shedding has been observed, no conclusive evidence supports active virus transmission to contacts. For example, 8.4% of family members in contact with T-VEC patients reported experiencing cold sore symptoms. However, these symptoms are mild, unconfirmed as OV infections, and do not pose significant clinical concerns. The results suggest that although viral shedding occurs, the risk of transmission remains minimal when appropriate safety measures are followed.^315,435^ Furthermore, special safety precautions may be needed when specific viruses are used in immunocompromised patients. Genetic modifications in OVs, especially those containing recombinant DNA elements, raise concerns about possible recombination with wild-type viruses, increasing the complexity of their safety profile.

Viral shedding during OV treatment has been documented in several clinical trials, primarily in blood, serum, and urine.^449^ Other fluids and tissues, such as saliva, oral swabs, cerebrospinal fluid, peritoneal lavage fluid, and injection sites, have also shown evidence of viral shedding.^450^ Infectious viruses shed during treatment can disseminate throughout the patient’s body and to individuals most likely to come into contact with these fluids, particularly family members and healthcare workers. Although the shed viruses observed in studies were highly attenuated and limited, posing minimal harm, the doses of OVs used in cancer treatments are generally too low to cause significant shedding. To minimize environmental viral shedding further, healthcare provider exposure should be strictly controlled during OV administration. Continuous monitoring of safety protocols and OV behavior in the body is crucial to ensure the safety and efficacy of these therapeutic agents.

### Antiviral immunity

The clinical effectiveness of OVs is often hampered by both the innate and adaptive immune responses targeting the viruses.^451,452^ These immune responses, particularly antiviral immunity, can accelerate the clearance of OVs from the host, thus significantly reducing their therapeutic efficacy.^453^ Both intravenous and intratumoral delivery of OVs are susceptible to these immune responses. The activation of the antiviral immune response inhibits viral replication, thereby undermining the intended tumor-targeting effects of OVs.^435 Th^is challenge underscores a key issue in OVT: how to manage antiviral immunity while ensuring sustained viral replication for antitumor activity.^454^ Achieving this balance is crucial for improving the therapeutic success of OVT, as antiviral immunity can hinder viral persistence while simultaneously promoting immune responses that aid in tumor eradication.

A crucial aspect of OVT in cancer therapy is the complex interaction between the virus and the host immune system. Rapid activation of antiviral responses is vital for suppressing viral replication. This involves the humoral immune response, including neutralizing antibodies and complement system activation. The swift response of innate immune cells, such as neutrophils and NK cells, also significantly contributes to overall resistance. Together, these factors rapidly suppress viral replication.^455^ This challenge is particularly pronounced with OVs derived from well-characterized viruses such as HSV or Ad. In such cases, patients may possess cross-reactive antibodies from prior exposure to these viruses, which can substantially hinder viral replication.^456^ Furthermore, patients with advanced-stage cancer are more likely to develop neutralizing antibodies after multiple OV treatments, diminishing the therapeutic efficacy of OVT. The presence of these antibodies presents a major obstacle to repeated OV administration by facilitating the premature clearance of OVs, thereby reducing their antitumor effects. Therefore, early modulation of these immune responses is necessary to increase viral replication and prolong viral persistence in the host, improving the likelihood of a successful therapeutic outcome.^457 Se^veral strategies have been proposed to manage antiviral immune responses in OVT. Immunosuppressants, such as cyclophosphamide, can enhance viral replication by suppressing the host immune response.^458^ However, the use of such agents must be approached with caution, as excessive immune suppression could inadvertently compromise the antitumor immune response, which is essential for sustained tumor regression.

**Fig. 5:**
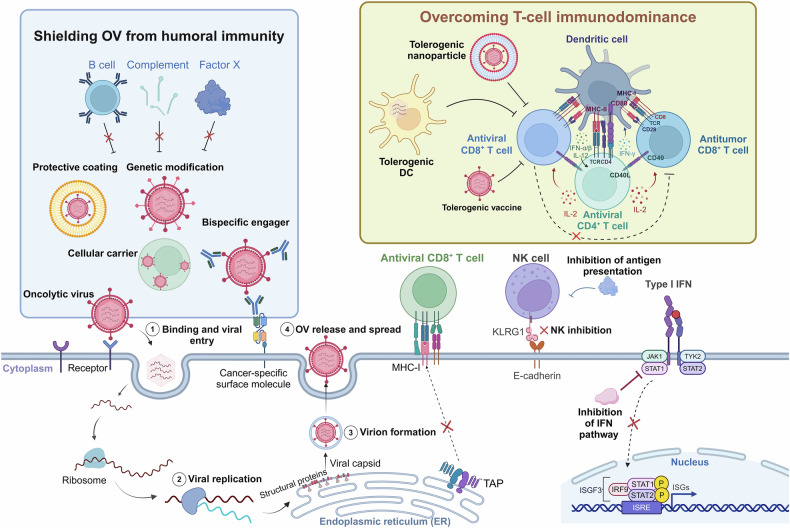
Strategies to circumvent antiviral immunity in oncolytic virotherapy. The therapeutic efficacy of OVs is often limited by host immune defenses, including humoral, innate, and adaptive immune responses. Strategies to overcome these challenges include viral encapsulation in protective coatings, stem cell carriers, and capsid modifications to evade preexisting antibodies. Bispecific engagers enhance viral delivery by targeting both neutralizing antibodies and tumor cells. To bypass innate immune barriers, approaches such as inhibiting IFN signaling, modulating NK cells, and utilizing tolerogenic DCs or nanoparticle delivery systems are employed. These combined strategies reduce immune clearance, promote viral replication, and enhance OV antitumor efficacy. ISG interferon-stimulated gene, MHC major histocompatibility complex, NK natural killer, TAP transporter associated with antigen processing, DCs dendritic cells. Created with BioRender.com

While antiviral immunity is generally considered detrimental to OVT, it can also provide inherent anticancer benefits. In certain contexts, antiviral immunity can recruit immune cells to the TME and reverse its immunosuppressive nature.^455,459,460^ Chiocca et al. demonstrated that antiviral responses, although limiting viral replication, can also trigger antitumor immune responses. This occurs through the recruitment of immune cells, particularly NK cells, to the tumor site.^461^ Such immune modulation can help mitigate the immunosuppression commonly observed in tumors, making antiviral immunity a double-edged sword in OVT. Therefore, the success of OVT depends on achieving an optimal balance between antiviral and antitumor immune responses. Researchers have focused on modulating the host immune system to maximize antitumor responses while minimizing antiviral responses and virus clearance (Fig. 5).^462^

These strategies can be divided into three main categories. The first strategy involves shielding OVs from humoral immunity. Protective coatings (e.g., liposomes, nanovesicles)^463–465^ and cellular carriers (e.g., mesenchymal stem cells)^466^ protect OVs from neutralizing antibodies and complement activation, enhancing viral persistence and tumor targeting. Genetic modifications of the virus, such as altering surface epitopes, can prevent immune system recognition and neutralization.^467^ Inhibition of the type I IFN signaling pathway via pharmacological agents can promote viral replication by suppressing antiviral responses.^468^ Moreover, modulating NK cell activity by initially depleting and subsequently reactivating these cells optimizes viral replication and enhances tumor-targeting immune responses.^469^ Furthermore, the use of nanoparticles to deliver viral antigens redirects the immune response from viral to tumor-specific antigens.^470^ Tolerogenic DCs and CAR-T-cell therapies enhance immune responses by redirecting the immune system toward the tumor, improving the synergy between OVs and antitumor immunity.^471,472^

In conclusion, overcoming antiviral immunity through protective coatings, genetic modifications, and immune modulation strategies is essential for enhancing the clinical outcomes of OVT. By combining these approaches, OVs can be shielded from immune clearance, allowing sustained viral replication and enhanced antitumor activity. However, a key challenge remains optimizing these strategies to ensure that immune suppression does not compromise the overall antitumor immune response. Effective virotherapy requires a careful balance between protecting OVs from immune clearance and promoting a robust tumor-specific immune response.

### Regulatory challenges

OVs, as promising cancer therapies, face unique regulatory challenges in clinical development because of their nature as live replicating viruses and their ability to be administered via intratumoral methods. These properties make traditional evaluation methods less suitable for OVs, particularly in terms of clinical trial endpoints, pharmacokinetics, dosing, and scheduling.^473^ First, the pharmacokinetic and pharmacodynamic properties of OVs differ markedly from those of conventional anticancer agents. OVs replicate selectively within tumor cells, often triggering complex immune responses and delaying clinical outcomes. Therefore, standard clinical trial endpoints and evaluation criteria, such as the Response Evaluation Criteria in Solid Tumors (RECIST), may be inadequate for accurately assessing OV efficacy. Alternative endpoints and extended treatment beyond conventional progression criteria should be considered. For example, in T-VEC clinical trials, over 50% of patients exhibited delayed responses, indicating that early-emerging lesions may reflect delayed therapeutic effects rather than true disease progression.^40^ The development of effective dosing strategies for OVs involves multiple considerations, including the maximum achievable concentration under current good manufacturing practices (GMPs), production capacity, viral immunogenicity, preexisting neutralizing antibodies, tumor histology, and transgene expression. Additionally, adjustments based on tumor volume and the daily maximum safe dose, considering viral pathogenicity, are needed. Preclinical animal model data may be limited if the selected models fail to support efficient viral replication.^474^ Moreover, selecting appropriate control groups in randomized studies presents challenges, particularly owing to the absence of approved standard-of-care intratumoral injection therapies. It is also essential to determine whether the systemic effects of OVs should be evaluated. The use of OVs in the neoadjuvant therapy setting may facilitate the evaluation of their early therapeutic potential.^475^ At this stage, the tumor burden is typically reduced, and the immune suppression and evasion pressures within the TME are diminished.^473^ This provides a window in which tumors are smaller and immunosuppressive mechanisms are underdeveloped, enabling more efficient OV infection and oncolysis. Finally, the clinical development of OVs requires stringent quality control and testing procedures, including assessments of viral identity, purity, potency, stability, mycoplasma contamination, and tumorigenic potential. As OVs are derived from biological materials, adherence to aseptic techniques and good manufacturing practices is essential. Early engagement with regulatory authorities and meticulous clinical trial design are critical for defining appropriate clinical trial endpoints.^476^

### Personalized medicine in OVT

The emergence of personalized medicine has revolutionized cancer treatment by focusing on tailoring therapies to individual genetic, molecular, and immunological profiles. OVs, genetically engineered or naturally occurring viruses that selectively infect and lyse cancer cells, represent a promising frontier in this evolving paradigm. However, the multifactorial nature of OV-tumor interactions presents a significant challenge. Identifying a universal biomarker or set of biomarkers to predict OV efficacy is unlikely due to the complexity of tumor biology and viral‒host interactions.^387^ This complexity is particularly evident in the variable responses across different cancer types, with different OVs showing varying levels of success depending on the tumor’s genetic, immunological, and metabolic characteristics.^477^ For example, T-VEC has demonstrated considerable promise in melanoma, inducing tumor regression in injected lesions while stimulating systemic antitumor immunity.^473^ These findings highlight the importance of selecting OVs that can effectively interact with the TME to maximize their therapeutic potential.

Precision medicine optimizes OVT by leveraging comprehensive patient data, including genetic, molecular, and immunological profiles. This enables the customization of OVs and combination strategies, optimizing antitumor responses and harnessing the synergistic potential of therapies.^34^ A significant advancement in this area is the integration of artificial intelligence (AI) and proteomics, which have transformed the design of personalized cancer therapies.^478^ Proteomics helps identify tumor-specific protein markers that influence viral infection, immune evasion, and tumor progression. These markers provide insights that genomic data alone cannot reveal.^479–481^ For example, in glioblastoma, RNA-based OVs have shown a correlation between viral sensitivity and deficiencies in IFN response pathways, emphasizing the need to tailor OV selection on the basis of molecular characteristics that extend beyond genomic mutations. Such insights underscore the importance of integrating both genomic and proteomic data for a more comprehensive understanding of tumor vulnerabilities. AI plays a critical role in analyzing large proteomic datasets, allowing for the identification of patterns that link protein expression with OV efficacy.^482 Ma^chine learning algorithms predict interactions between viral and tumor proteins, enabling the personalized selection of OVs that are most likely to trigger successful oncolysis by targeting specific tumor markers.^483^ Additionally, AI can model how the TME affects OV distribution and immune responses, optimizing viral delivery and addressing challenges such as immune-mediated clearance or elevated interstitial fluid pressure within tumors.

Virus engineering is central to enhancing the specificity and efficacy of OVT. AI-driven approaches, such as AlphaProteo models, utilize deep learning algorithms to predict and design viral proteins with high binding affinity to tumor-specific receptors, which are often upregulated in various cancers. This targeted design ensures that viruses selectively infect cancer cells while minimizing off-target effects. Furthermore, AI can be employed to conduct molecular dynamics simulations to predict the binding energy between viral proteins and tumor receptors.^484^ These simulations enable the screening of the best viral strains with the highest affinity for cancer cells, ensuring that viral oncolysis is more efficient and precise.^485^ By refining viral engineering via these advanced AI techniques, it is possible to develop more effective and specific OVs for various cancer types.^486^ In addition to virus engineering, AI-driven models can simulate how the TME affects OV distribution and the immune response.^487^ These models incorporate factors such as protein-mediated immune suppression and viral replication dynamics within tumors.^488,489^ By modeling these interactions, AI can guide the design of treatment strategies that overcome immune resistance and optimize OV delivery.^490^ This step is critical for ensuring the effectiveness of viral therapies in immunosuppressive environments, such as those present in certain solid tumors.

To further refine OVT, integrating multi-omics, including genomics, proteomics, and metabolomics, provides a holistic view of the molecular landscape of tumors. While genomic sequencing offers insights into tumor drivers, proteomics delves deeper into how protein expression and immune evasion mechanisms influence OV resistance. AI facilitates the integration of these diverse datasets, enabling the personalized selection of OVs that target both genetic mutations and tumor-specific protein profiles. This comprehensive, data-driven approach enhances the ability to predict therapeutic outcomes, optimize treatment regimens, and address the heterogeneity of tumor biology.^487^ By employing a multi-omics approach, personalized OVT becomes more precise, enabling the selection of the most appropriate virus for each patient’s unique tumor profile.^491^ This approach optimizes therapeutic outcomes, maximizes viral oncolysis, and holds the potential to overcome existing challenges in the treatment of various cancers.

In addition to genetic and immune factors, patient-specific variables, such as sex-based differences, must be integrated into OVT. Biological sex significantly influences immune responses, with females generally exhibiting stronger antiviral immunity than males.^492,493^ These differences have significant implications for OVT, as they may affect patients’ responses to viral infections and antitumor immune activation. As highlighted by previous studies on sex differences in biomedical research, including both male and female animal models in preclinical OV studies, sex differences are essential for understanding how sex influences therapeutic outcomes.^494^ Moreover, clinical trials must consider sex as a key variable, helping to refine OV dosing and scheduling, thereby maximizing efficacy across sexes. Considering sex-based differences can further personalize OVT and improve treatment outcomes across diverse patient populations.^495^

Historically, most clinical studies have evaluated individual OVs primarily on the basis of availability rather than rigorous comparative efficacy analysis.^496^ This selection bias may have constrained the identification of more potent OVs and hindered overall progress in optimizing OV-based therapies. Notably, few preclinical studies have systematically compared the antitumor efficacy of different OVs, despite substantial potential benefits. Future studies should utilize standardized in vitro and in vivo models, along with advanced computational simulations, to systematically evaluate and rank OVs for specific patient populations.^497^ Multi-omics data can reveal tumor susceptibility factors, including receptor density and immune evasion mechanisms, which can be integrated into a “viral sensitivity index” (VSI). This index offers a quantitative framework to evaluate tumor responsiveness on the basis of molecular and immunological criteria, enabling more precise OV selection for individual patients.

To bridge the gap between preclinical findings and clinical success, patient-derived tumor xenograft (PDX) and patient-derived organoid (PDO) models are crucial for evaluating OVT.^498^ These models preserve tumor heterogeneity and architecture, enabling more accurate assessments of OV efficacy. By employing PDX and PDO systems, researchers can more accurately evaluate OV interactions with the TME, improving predictions of patient-specific responses and refining clinical trial inclusion criteria.^499^ In addition, PDO models, three-dimensional cultures derived from patient tumors, provide an important platform for studying OV dissemination, tumor–virus interactions, and immune modulation. Compared with traditional 2D cultures, PDOs retain the molecular and structural characteristics of the tumor and its microenvironment, offering a more accurate representation of OV behavior.^500^ Combining PDX and PDO models enables the assessment of different OV strains, guiding personalized therapies and evaluating OV combinations with ICIs or chemotherapy for potential synergies.

The future of OVT lies in the integration of multi-omics data, big data analytics, and synthetic biology. Leveraging these technologies, researchers can develop customized OVs tailored to individual genetic and molecular profiles while dynamically adapting to changes in the TME. This strategy represents a shift from a one-size-fits-all model to a genuinely personalized therapeutic paradigm. The development of the VSI, along with advanced models such as PDXs and PDOs, could provide nuanced insights into tumor responsiveness to specific OVs, guiding the optimal selection of viral agents. Simultaneously, considering microbiome influences and employing advanced preclinical models could increase the accuracy of clinical outcome predictions and further optimize OVT for patient-specific applications. By integrating these innovative strategies, the full therapeutic potential of OVT can be more effectively realized in personalized cancer care.

## Future prospects and conclusion

**Fig. 6:**
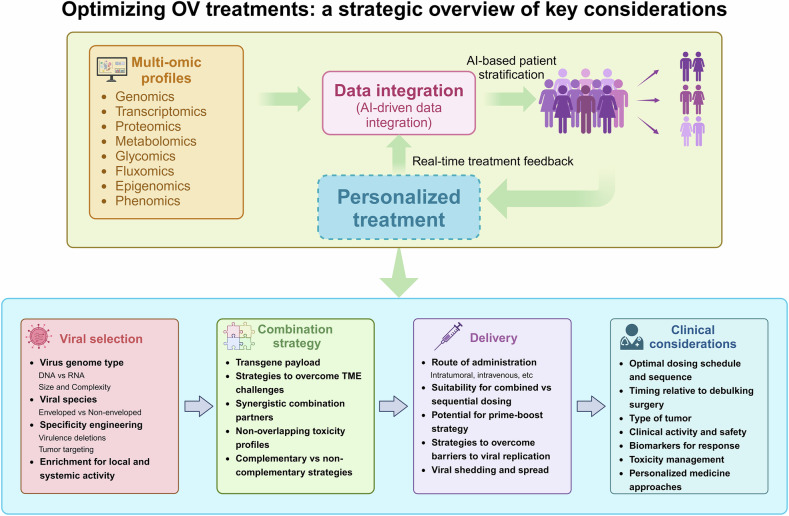
Optimizing OV treatments: a strategic overview of key considerations. The optimization of OVT begins with multi-omics profiling and AI-driven data integration, which stratify patients on the basis of unique genetic, epigenetic, immune, and metabolic characteristics. This stratification informs personalized treatment strategies, guiding virus selection, combination therapies, and delivery strategies tailored to individual needs. Once patients are stratified, virus selection is based on factors such as genome type (DNA vs RNA), tumor tropism, and engineered modifications for enhanced oncolysis. Additionally, combination therapies incorporating ICIs, chemotherapy, radiotherapy, and other targeted therapies are designed to overcome immune-suppressive barriers in the TME and enhance therapeutic synergy. Effective delivery strategies ensure efficient viral replication while overcoming barriers, including immune evasion and viral spread. Finally, clinical considerations such as optimal dosing, tumor type, and safety monitoring are critical to ensure that personalized treatment plans are both effective and safe, maximizing therapeutic benefits while minimizing adverse effects. Created with BioRender.com

OVT has emerged as a transformative modality in cancer immunotherapy, leveraging a dual mechanism by directly lysing tumor cells and simultaneously stimulating systemic antitumor immune responses. Despite its therapeutic potential, the clinical translation of OVT faces several challenges, including viral engineering, delivery optimization, tumor heterogeneity, and rational therapeutic combinations, which must be addressed to integrate it into personalized cancer treatment regimens (Fig. 6).

Cutting-edge gene editing systems and synthetic biology platforms enable the precise design, synthesis, and modification of viral genomes, representing a key approach for optimizing the therapeutic precision and oncolytic efficacy of OVT. For example, CRISPR-Cas9^501^ and synthetic biology techniques^502^ enable precise modifications to viral genomes, facilitating the development of multifunctional OVs capable of delivering therapeutic payloads, such as immunostimulatory cytokines, ICIs, or even gene-editing tools.^503–505^ Furthermore, advancements in capsid engineering to overcome tumor resistance mechanisms and enhance viral entry into tumor cells will substantially improve OV efficacy, especially in hard-to-treat cancers.^506^

Tumor heterogeneity remains a substantial obstacle for the widespread application of OVT.^507^ Variability in tumor genomic architectures and phenotypic states across patients results in divergent therapeutic responses to OVT. Personalized medicine plays a crucial role in overcoming this challenge by tailoring OVT on the basis of individual patient profiles. Genetic and immune profiling enables clinicians to customize OVT according to specific tumor characteristics, which ultimately improves clinical outcomes. Next-generation personalized OVT strategies integrate multi-omics profiling,^508,509^ patient-derived organoid drug sensitivity testing^510–512^ and circulating tumor DNA monitoring to establish predictive frameworks.^513^ These frameworks enable the precise stratification of patients for OVT monotherapy and combination therapy regimens, improving clinical outcomes.^514^

Efficient delivery of OVs to tumors remains a critical challenge in clinical translation. Intratumoral injection, although effective for localized tumors, is technically challenging and often impractical for deep-seated or metastatic lesions. Systemic delivery through intravenous administration offers broader applicability but faces significant obstacles, including rapid immune clearance and limited viral stability in circulation. To address these limitations, future strategies could employ nanoparticles,^515,516^ mesenchymal stem cells,^517^ or other biomaterials^518^ to encapsulate or shield OVs from immune surveillance, thereby improving therapeutic efficacy. Moreover, viral engineering strategies aimed at evading preexisting immunity, including capsid protein modifications or the selection of immune-evasive viral strains, could further optimize systemic OV delivery. Moreover, beyond intravenous injection, alternative routes such as portal vein administration may offer more direct access to liver and abdominal tumors, potentially improving localized viral delivery.^519^ These combined strategies aim to increase OV stability, reduce immune-mediated clearance, and optimize their therapeutic impact across a broader range of tumor types and locations.

Despite substantial progress, regulatory and manufacturing barriers persist. Long-term safety evaluations, high production costs, and the requirement for frozen storage complicate the routine clinical use of OVs.^520^ The development of lyophilized formulations and streamlined manufacturing processes will be critical to reduce costs and improve clinical accessibility. Regulatory frameworks must also evolve to accommodate the distinct nature of live viral therapies, ensuring rigorous safety and efficacy assessments while facilitating expedited clinical approval.

One of the most promising aspects of OVT is its potential to be combined with other therapeutic modalities to maximize treatment efficacy. The combination of OVT with immune-based therapies holds significant promise. For example, pairing OVT with ICIs, including anti-PD-1 or anti-CTLA-4 antibodies, can enhance immune cell infiltration and improve immunotherapy efficacy.^521,522^ The integration of OVs with ACT, such as CAR-T-cell therapy or invariant natural killer T-cell (iNKT) therapy, can increase T-cell infiltration and persistence at tumor sites, which is often limited in solid tumors.^285,523^ Additionally, incorporating epigenetic-modifying drugs can further sensitize tumors to viral oncolysis, strengthening the overall therapeutic response. Owing to the ability of OVs to remodel the TME, they can convert “cold” tumors (low immune infiltration) into “hot” tumors (immune-rich), increasing their susceptibility to ICIs and other immunotherapies. In addition to immunotherapy, OVT also shows promise when used alongside conventional chemotherapy and radiotherapy, as viral infection can increase tumors sensitivity to these treatments by inducing DNA damage and disrupting tumor resistance mechanisms. Future research should focus on optimizing the sequence, dosage, and combination strategies to fully exploit the synergistic potential of OVs in multimodal cancer therapy.

Ultimately, the future of OVT depends on its integration into personalized cancer treatment strategies. As the field evolves, OVs are likely to become part of a broader therapeutic arsenal, combined with chemotherapy, radiotherapy, immune-based therapies, and gene-editing technologies. The ability of OVT to induce tumor regression and enhance immune responses is critical for achieving durable, long-term remission, particularly in cancers resistant to conventional therapies. In this context, OVT has the potential to transform cancer treatment, offering patients more precise, effective, and less toxic options than traditional therapies do.

In conclusion, over the next decade, OVT will continue to evolve, driven by innovations in viral engineering, combination therapies, and personalized treatment strategies. As clinical trials progress and overcome existing challenges, OVT has the potential to become a cornerstone of precision oncology, providing new hope for patients with difficult-to-treat cancers. By integrating immuno-oncology approaches with genetic, epigenetic, and immune profiling, OVT could redefine cancer treatment paradigms, advancing toward the goal of providing tailored therapies that maximize efficacy while minimizing adverse effects.
